# Multi-scale optoacoustic molecular imaging of brain diseases

**DOI:** 10.1007/s00259-021-05207-4

**Published:** 2021-02-16

**Authors:** Daniel Razansky, Jan Klohs, Ruiqing Ni

**Affiliations:** 1 Institute for Biomedical Engineering, University of Zurich & ETH Zurich, Wolfgang-Pauli-Strasse 27, HIT E42.1, 8093 Zurich, Switzerland; 2 Zurich Neuroscience Center (ZNZ), Zurich, Switzerland; 3 Faculty of Medicine and Institute of Pharmacology and Toxicology, University of Zurich, Zurich, Switzerland; 4 Institute for Regenerative Medicine, Uiversity of Zurich, Zurich, Switzerland

**Keywords:** Brain, Neuroimaging, Optical imaging, Multi-spectral optoacoustic tomography (MSOT), Photoacoustics

## Abstract

The ability to non-invasively visualize endogenous chromophores and exogenous probes and sensors across the entire rodent brain with the high spatial and temporal resolution has empowered optoacoustic imaging modalities with unprecedented capacities for interrogating the brain under physiological and diseased conditions. This has rapidly transformed optoacoustic microscopy (OAM) and multi-spectral optoacoustic tomography (MSOT) into emerging research tools to study animal models of brain diseases. In this review, we describe the principles of optoacoustic imaging and showcase recent technical advances that enable high-resolution real-time brain observations in preclinical models. In addition, advanced molecular probe designs allow for efficient visualization of pathophysiological processes playing a central role in a variety of neurodegenerative diseases, brain tumors, and stroke. We describe outstanding challenges in optoacoustic imaging methodologies and propose a future outlook.

## Introduction

### Optoacoustic imaging and tomography as a new modality in neuroscience research

**Fig. 1:**
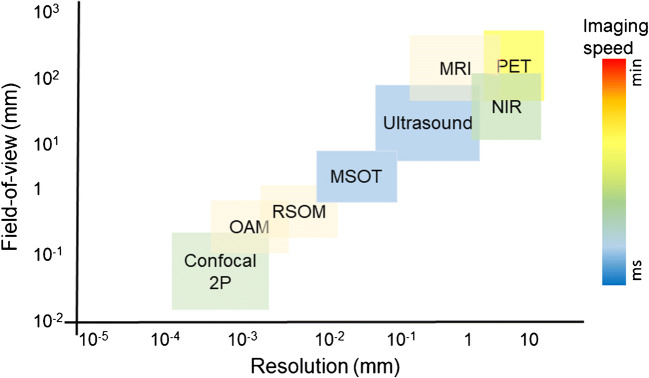
Resolution and field-of-view of different imaging modalities. 2P, two-photon; OAM, optoacoustic microscopy; MSOT, multispectral optoacoustic tomography; RSOM, raster-scan optoacoustic mesoscopy; NIR, near-infrared fluorescence imaging; PET, positron emission tomography; MRI, magnetic resonance imaging

Advances in imaging technology have led to tremendous breakthroughs in life sciences and biomedicine. In particular, cutting-edge neuroimaging tools have greatly aided in the understanding of brain organization while also being instrumental in studying and treating brain disorders [1–8]. Imaging studies in animal models of human disease have enabled dissection of disease mechanisms and identification of molecular targets toward the development of novel therapeutic strategies [9–11]. While significant progress has been made in understanding the molecular pathophysiology of most brain diseases, we are still far from linking information on molecules, gene regulatory events, and signaling pathways to the changes that occur over time on the tissue and organ level in an integrative way [12]. For example, while some pathological processes occur on short time scales or small spatial scales (cellular and sub-cellular scale), e.g., synaptic dysfunction, other processes, such as neurodegeneration, develop over years and decades before being manifested as gross histopathological changes. Imaging methods that can visualize and quantify several processes over prolonged durations in vivo are highly desired, but pose high demands on the sensitivity, field-of-view (FOV), depth of penetration, and spatial and temporal resolution of the imaging techniques. Moreover, the ability to capture the interplay between different cellular and molecular pathophysiological processes implies the use of methods providing highly multiplexed information. Figure 1 summarizes the performance of various brain imaging modalities with respect to their FOV, resolution, and imaging speed. State-of-the-art confocal and two-photon microscopies allow for the investigation of cellular processes with high spatial resolution *in vivo*. However, microscopic methods come with the price of a limited (sub-millimeter) depth penetration in mammalian brains, a small FOV, and a high degree of invasiveness. Non-invasive optical imaging techniques, such as near-infrared fluorescence imaging (NIRF) and tomography, exhibit high sensitivity and can be used in combination with imaging probes to observe biological processes at the cellular and subcellular level, deep in rodent brains [13, 14]. However, these techniques exhibit poor spatial resolution in the 1 mm range due to intense photon scattering in living tissues [15]. Magnetic resonance imaging (MRI) and X-ray computed tomography (CT) are routinely employed for clinical diagnostics and achieve whole-brain coverage and excellent anatomical contrast, making those techniques capable of characterizing gross morphological changes during disease progression [7, 16, 17]. However, those modalities come with low sensitivity to cellular and sub-cellular events. In contrast, positron emission tomography (PET) and single-photon emission computed tomography (SPECT) provide good detection sensitivity but at the expense of low spatial resolution and only brief observation windows due to decaying radioactive isotopes. In particular, functional ultrasound imaging is emerging as a powerful brain interrogation tool in rodents [18]. Transcranial-focused ultrasound is further explored for treating certain neurological and neurodegenerative conditions [19–21], yet highly sensitive detection of molecular events remains challenging with ultrasound imaging, which limits imaging applications to the vascular space where most pathological processes remain inaccessible.

Optoacoustic (OA) imaging, also termed photoacoustic, is an emerging tool in biomedical research providing a variety of complementary benefits to other imaging modalities in terms of contrast, penetration depth, and spatial and temporal resolution. OA relies on the photophonic phenomenon first described by Tainter and Bell in 1880 where intermittent light radiation is absorbed by molecules and converted into heat, leading to instantaneous thermoelastic expansion and induction of broadband pressure waves [22]. It has yet taken more than a century of technological progress bringing about intense pulsed laser sources, sensitive broadband ultrasound arrays, fast digitization electronics, and efficient image reconstruction algorithms, to enable practical biomedical OA imaging systems [23, 24]. Nowadays, tunable laser sources in the near-infrared range, where tissue optical absorption is reduced, are commonly employed for in vivo imaging applications, thus allowing for deep tissue penetration of the excitation photons. In this way, distribution of tissue chromophores and photoabsorbing agents can be rendered tomographically via single transducer scanning or parallelized OA signal detection with arrayed probes accompanied by suitable image reconstruction algorithms, e.g., based on back-projection or model-based inversion approaches [25, 26].

**Fig. 2:**
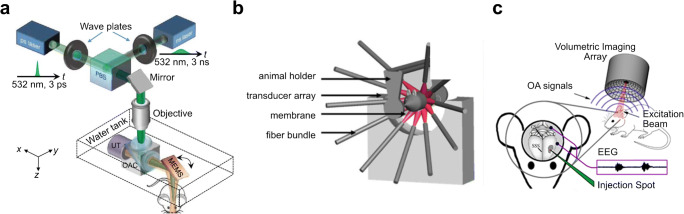
Examples of optoacoustic imaging setups used for molecular interrogation of brain diseases. **a** Optoacoustic microscopy OR-PAM. Adapted with permission from [25]; **b** Cross-sectional multi-spectral optoacoustic tomography (MSOT) imaging setup. Adapted with permission from [207]; **c** Volumetric MSOT. Adapted with permission from [29]

OA methods are ideally suited for functional and molecular in vivo interrogations at different scales, from single cells to whole organisms. For instance, optical-resolution optoacoustic microscopy (OR-OAM) uses scanning of focused optical excitation along the tissue surface thus attains high-resolution imaging of the mouse cortical areas at single capillary resolution [25] (Fig. 2a). Small animal scanners use instead tomographic data collection with partial- or full-ring concave transducer arrays to render cross-sectional reconstructions [26] or whole-body 3D scans by means of spiral volumetric optoacoustic tomography (SVOT) [29]. In addition, multi-spectral optoacoustic tomography (MSOT) can sensitively differentiate between different tissue chromophores or extrinsically administered probes via spectroscopic analysis to provide multiplexed information from the same animal (Fig. 2b) [27]. Fast repetition rate laser sources have been developed to enable data acquisition of cross-sectional images with high frame rates thus follow fast biological processes dynamically [28]. Further technical advances focus on real-time recording of true 3D spectroscopic data from the whole mouse brain in action [29, 30] (Fig. 2c). The combination of optical excitation with ultrasonic detection offers unique benefits for investigating the vascular system in model systems: (1) ultrasonic detection is not sensitive to light scattering; thus, the technique can provide rich optical contrast while extending the effective penetration compared to conventional optical microscopy methods by at least an order of magnitude (> 1 cm); (2) various OAM and MSOT implementations can achieve scalable imaging in the living brain, from capillary resolution in up to 1 mm deep cortical areas to whole mouse brain observations with sub-200 μm resolution [25, 30, 31]; (3) OA provides label-free visualization of multiple cerebral hemodynamic parameters [29, 32] along with highly sensitive spectroscopic differentiation of extrinsic labels [27, 28]. Simultaneous multi-parametric imaging of hemoglobin concentration, blood oxygenation, and blood flow, as well as the micro-regional cerebral metabolic rate of oxygen, is possible by means of multi-parametric OAM [33, 34]. As a result, OA techniques effectively bridge the gap between microscopic and macroscopic brain imaging realms and are uniquely endowed with high-resolution, fast, multiscale, and multiplex imaging capacities in small-animal organisms in vivo.

### Endogenous contrast for structural and functional imaging

**Fig. 3:**
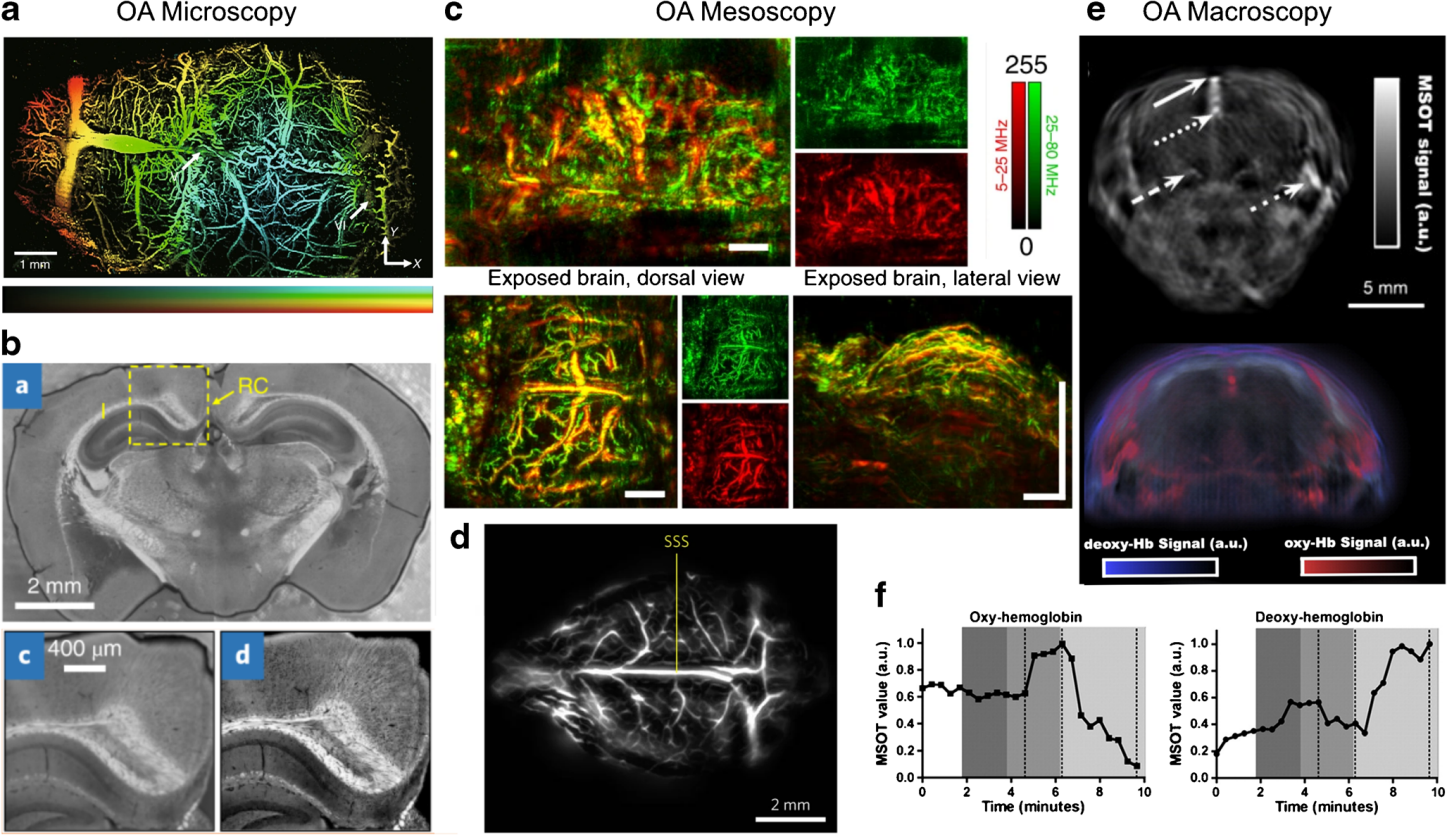
OA imaging of structural and anatomical information in small animal brain. **a** In vivo super-resolution localization optoacoustic microscopy (OAM) of a mouse brain using intrinsic contrast of red blood cells—depth and amplitude are color-encoded. Adapted with permission from [37]. Superior sagittal sinus (SSS) and transverse sinus are labeled with white arrows; **b** 300-μm-thick slices of the mouse brain cerebrum myelin imaged using mid-infrared (MIR)-OAM [39]. An ultraviolet-localized image is shown at the bottom right, exhibiting higher spatial resolution; **c** Raster-scan optoacoustic mesoscopy (RSOM) images of the mouse brain (dorsal and lateral views are shown with skin or skullcap removed). Scale bars, 1 mm. Adapted with permission from [36]; **d** Single-impulse panoramic photoacoustic–computed tomography (SIP-PACT) of vasculature in the mouse brain cortex. Adapted with permission from [37]; **e** In vivo MSOT imaging of the mouse brain showing single wavelength (800 nm) anatomical image, along with spectrally unmixed distribution of deoxy- and oxyhemoglobin; **f** Dynamics of cerebral blood oxygenation following carbon dioxide challenge imaged with MSOT (white, normal air; dark gray, 10% carbon dioxide; intermediate gray, 100% oxygen; and light gray, 100% carbon dioxide). Adapted with permission from [43]

**Fig. 4:**
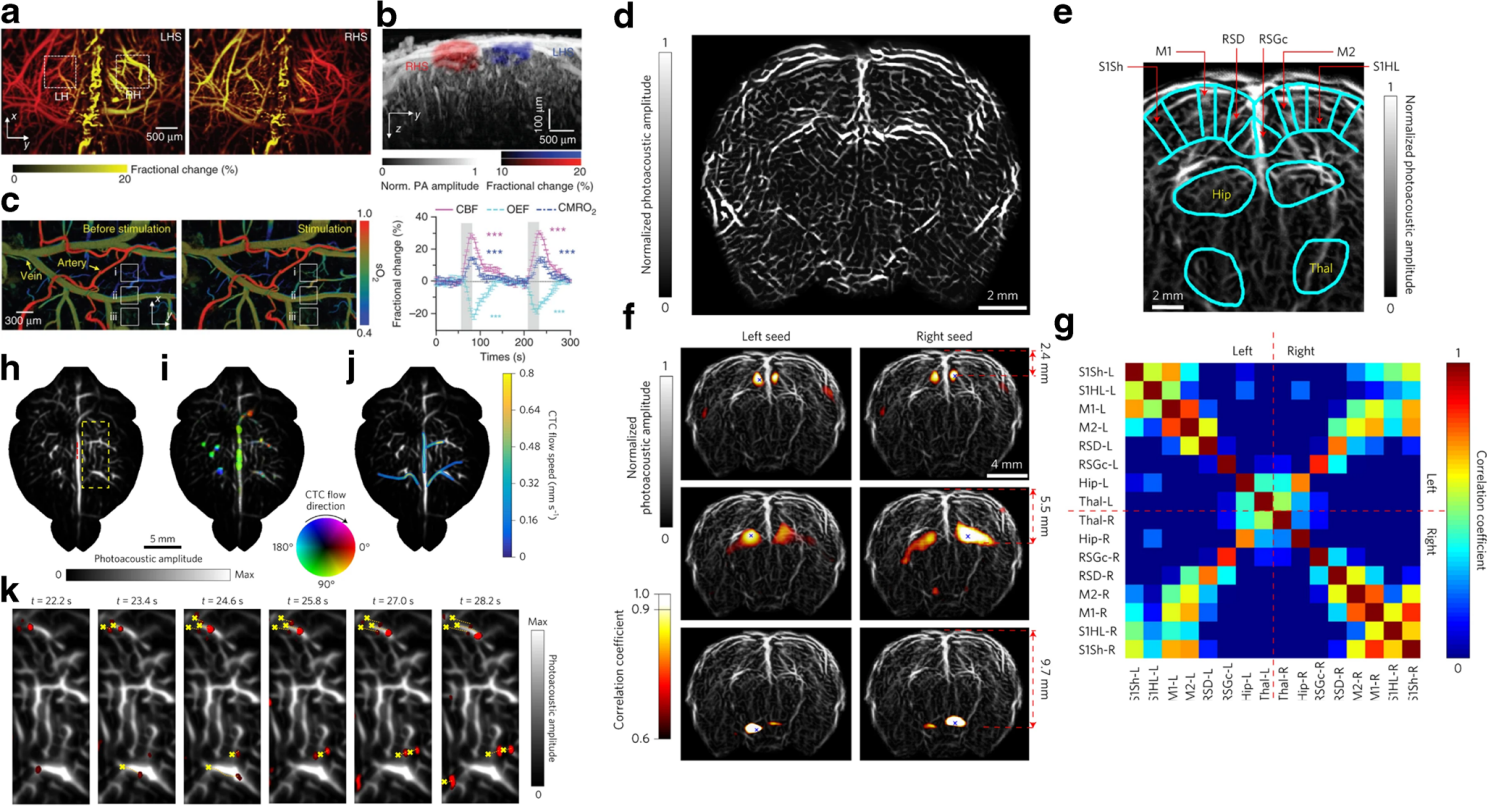
Functional imaging based on endogenous absorption contrast. **a-c** Optoacoustic microscopy of brain responses to electrical stimulations of the hindlimbs of mice. Adapted with permission from [25]; **d**-**g** Imaging of rat whole-brain functions using Single-impulse panoramic photoacoustic–computed tomography; **d** Whole-brain vasculature; **e** segmentations of different functional regions of the brain; **f** Seed-based functional connectivity analyses on both sides of the brain; **g** Correlation matrix of the 16 functional regions in **f**. Adapted with permission from [37]; **h–k** Single-impulse panoramic photoacoustic–computed tomography of the mouse cortex after injection of melanoma cancer cells, color represent the flow direction of circulating tumor cells (CTCs). Adapted with permission from [37]

In OA imaging, several contrast mechanism can be exploited to derive diverse information from tissues. Even though the soft tissue contrast of OA imaging might be inferior to other anatomical imaging modalities (e.g., MRI), OA microscopy and mesoscopy provide a unique ability for label-free visualization of the cortical vasculature in murine brains down to the capillary level [35–38] (Fig. 3a, c, d). For ex vivo samples, the high-resolution microtomy-assisted OAM can reach 400 nm resolution when imaging cell nuclei, blood vessels, axons, and myelin structures in paraffin or agarose-embedded mouse brain tissues [39, 40] (Fig. 3b). In biological tissues, major absorbers are melanin, water, lipids, and hemoglobin (i.e., deoxyhemoglobin, Hb and oxyhemoglobin, HbO_2_), which provide endogenous absorption contrast. Spectroscopic (MSOT) imaging (Fig. 3e–f) of hemoglobin has been employed in numerous applications aimed at attaining real-time readouts on the total hemoglobin concentration, blood oxygen saturation, cerebral blood volume (CBV), and oxygen metabolism [41–43]. By using sparsely distributed flowing microbeads and droplet, localization-based OAM [38] (Fig. 3a) and tomography [35, 37, 44–46] have been developed, which enabled in vivo micro-angiography of deep brain vasculature at 20 μm resolution, further providing cerebral blood flow (CBF) readouts. While functional MRI (fMRI) based on blood oxygen level-dependent (BOLD) signal is widely used for studying brain activity under resting state and stimulus-evoked conditions in humans and rodents [23, 29, 47–49], due to its fast imaging capacity, hemodynamic MSOT imaging is similarly establishing itself as a surrogate for brain activity in small animal models, correlating robustly with electroencephalogram (EEG) readouts [29, 31, 49–52]. Yao et al. demonstrated using OAM of brain responses to electrical stimulations of the hindlimbs of mice (Fig. 4a–c) [25]. Li et al. showed imaging of brain stimulation using snapshot photoacoustic topography through an ergodic relay for high-throughput imaging of optical absorption (Fig. 4d–g) [53]. Ovsepian et al. demonstrated the feasibility of imaging diverse spectroscopic endogenous contrast in the mouse brain ex vivo [54].

### MSOT probes for molecular imaging

The advent of new MSOT probes (Table 1), e.g., those based on small-molecule near-infrared dyes (e.g., cyanine dyes, squaraines, porphyrin derivatives), plasmonic (e.g., gold, single-walled carbon nanotubes), and polymer nanoparticles has facilitated their use for unspecific vascular contrast enhancement and conjugated to targeting moieties and enzymatic sensors [55–57]. In particular, organic dyes allow for stable labeling making them ideal for longitudinal studies. Small-molecule dyes currently used for MSOT imaging are also fluorescent, aiding ex vivo validation of biodistribution of probes in whole organs and tissue sections by using NIRF and fluorescence microscopy. In addition, nanoprobes have been developed for labeling and longitudinal tracking stem cells evaluating therapeutic treatment effects in stroke and brain injury models [58–66]. Li et al. demonstrated imaging of bone mesenchymal stem cells labeled with Prussian blue nanoparticles in a brain injury mouse model (Fig. 6c–e) [64]. Probes with theranostic potential have also been developed such as graphene oxide (rGO)-loaded plasmonic gold nanorod vesicle [67], and polymer nanoparticle conjugated with c-RGD peptide [68].

**Table 1:** Optoacoustic contrast

Endogenous contrast	Category	Application
Deoxy/oxyhemoglobin		Functional, metabolic [23, 30, 49, 82, 193]
Melanin		CTC in brain [37, 54]
Lipid		Myelin [40, 194, 195]
Indocyanine green (ICG)*	Organic dye	Brain tumor [196]
IRDye800cw		Tumor [192]
AOI987, CRANAD-2, CDA-3, Congo red		Amyloid-beta [100, 118, 119, 126]
BODIPY		[159]
*N*,*N*-dimethylaniline (RPS1)		Brain copper2+ accumulation [129]
dipicrylamine		Voltage response in epilepsy [157]
MMPsense680	Peptide	Inflammation in stroke model [142]
Peptide ligand cRGD		Brain tumor [90]
Prussian blue-poly(l-lysine) NP	NIR I nanoprobe	Track stem cell and brain injury [61–64, 197]
Ca-pSiNPs-ICG, MoS(2)-ICG		[65] Brain tumor [104]
iron NP		Image-Guided Surgery [89]
Carbon nanotubes		Brain tumor [94, 96, 162, 198, 199]
Gold NP		Brain tumor [69, 89, 91, 98, 200]
Quantum dot		Image-guided photothermal therapy [95, 97], Brain tumor [109]
Cu _2- x_ Se NP		Blood brain barrier [201]
H_2_O_2_-responsive liposomal NP		Inflammation [87]
1-RGD		Brain tumor apoptosis, caspase-3 [202]
PBT; semiconducting polymer NP		Brain tumor [68, 91, 99, 107, 203–205], Vasculatures [88, 206] ;
rsOAPs; BphP1; DrBphP-PCM, iRFP	Bacterial phytochrome	Brain tumor [69, 71–73]
iGECI, GCaMP, CaMPARI	GECI	Calcium imaging [70, 76–78]
Voltage gated	GEVI	Calcium imaging [80]

*FDA approved, *NP* nanoparticle

**Fig. 5:**
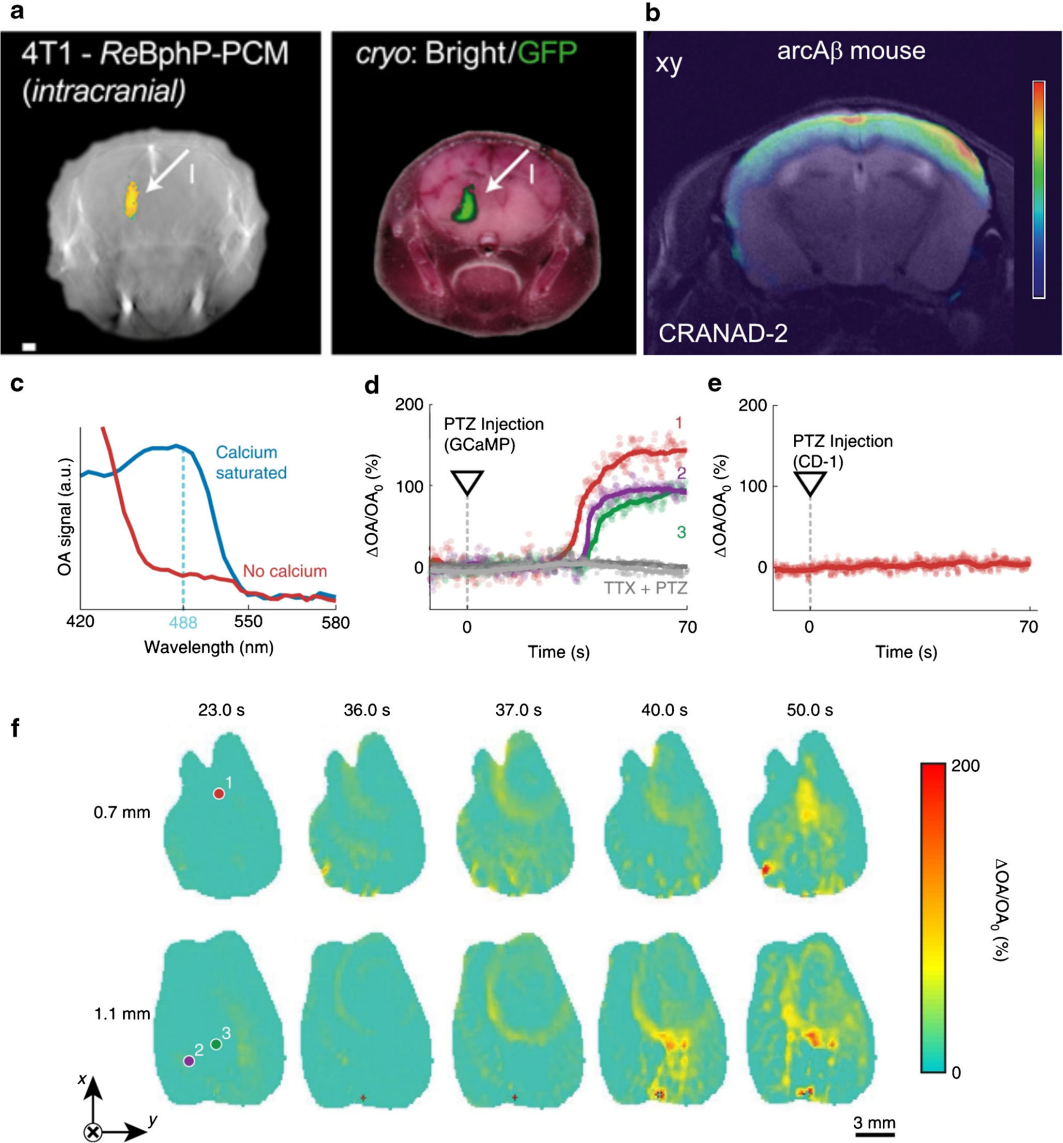
Molecular (contrast-enhanced) MSOT imaging at the whole-brain level. **a** 4T1 cells (0.7 × 10^6^ injected intracranially) stably expressing ReBphP-PCM imaged using MSOT at a depth of 3.6 mm in the brain (arrow I) immediately after injection. Adapted with permission from [71]; **b** volumetric MSOT imaging of amyloid-beta plaque in Alzheimer arcAβ mouse brain using amyloid binding probe CRANAD-2, showing higher signal retention in the cortical areas compared to age-matched wild-type mice. Adapted with permission from [120];** c-f** whole-brain functional optoacoustic neuro-tomography (FONT) volumetric imaging of calcium waves induced by pentylenetetrazole (PTZ) injection into an isolated mouse brain model. Adapted with permission from [31]; **c** absorption spectrum of purified calcium-saturated (blue) and calcium-free (red) GCaMP6f proteins. **d** Time traces of the normalized FONT data. Gray traces represent tetrodotoxin (TTX) injected 180 s before PTZ (*t* = 0 s), abolishing the activation; **e** no signal change due to PTZ injection are detected in a control isolated CD-1 mouse brain not expressing GCaMP6f proteins; **f** temporal evolution of the relative signal changes (∆OA/OA_0_) in slices at depths of 0.7 mm and 1.1 mm in a GCaMP6f-expressing brain

Genetic reporter systems have also been developed to enable MSOT imaging of gene expression. Existing genetic sensors for non-invasive molecular imaging [69, 70], such as the pigment enzyme reporters, fluorescent proteins, chromoproteins, and photo-switchable proteins (e.g., rsOAPs, BphP1, DrBphP-PCM, iRFP [69, 71–73]), have provided excellent means for whole-brain MSOT imaging [74, 75]. Efforts have been recently made to develop genetically encoded calcium indicators (GECIs) [76–79], and voltage-gated indicators [80], as well as astrocyte- and neuronal-specific sensors [81]. Dean-Ben et al. demonstrated rapid imaging of calcium transients elicited by pentylenetetrazole in HuC:GCAMP5g zebrafish larvae using functional optoacoustic neuro-tomography (FONT) [82]. Gottschalk et al. further showed rapid, high-resolution 3D mapping of large-scale Ca^2+^ neuronal activity across the mammalian brain in models expressing genetically encoded calcium indicator GCaMP6f using FONT [31] (Fig. 5c–f). The recent advances in development of near-infrared-shifted NIR-GECO indicators [78] hold promise for mapping neural activity with lower background absorption levels and increased penetration depth, both with fluorescence-based and OA modalities.

### Application in elucidating brain diseases

#### Gliomas

Gliomas are the most prevalent type of primary malignant brain tumors and are associated with high mortality and morbidity. Conventional therapy comprises surgical resection, radiation, and/or chemotherapy, but the responses to the treatment regimens are only the modest and associated with adverse effects. Imaging techniques such as CT and MRI have been critically implicated in the development of precision medicine related to gliomas and other cancer types [83]. While useful for diagnosis, current anatomical imaging can neither adequately determine the most effective treatment regimen for individual patients nor reliably monitor treatment response. Thus, advanced imaging techniques that are capable of quantitatively interrogating glioma biology at the physiologic, cellular, and molecular levels are highly desired. Angiogenesis has been visualized using endogenous hemoglobin contrast in tumor models bearing U87MG [43, 67, 84] and orthotopic brain glioma [36, 53, 68, 85–88], where elevated signal levels were observed in the tumor regions. In addition to the diagnostic potential in tumor staging, MSOT imaging has been assisting imaging-guided surgery [89] for visualizing tumor margins using contrast agents, e.g., gold or other nanoparticles [68, 84, 90, 91], in evaluation of photothermal therapy [68, 90, 92, 93], pharmacological treatment [90, 94–100], and radiation therapy response [101].

The completeness of the surgical resection is a key factor in the prognosis of patients with brain tumors. Despite its known limitations, MRI using gadolinium-based contrast enhancement currently remains the gold standard for diagnosis and pre-surgical planning [102]. Several alternative optical methods have been suggested for intraoperative delineation of tumor margins, based either on intrinsic optical tissue properties or exogenous contrast agents. The location of the tumor can be tracked deep inside the brain with MSOT, as demonstrated by Deliolanis et al. on iRFP-labeled U87MG expressing glioblastoma tumor implants [103] and by Mishra et al. on 4T1 cell mouse mammary gland tumors expressing ReBphP-PCM [71] (Fig. 5a).

**Fig. 6:**
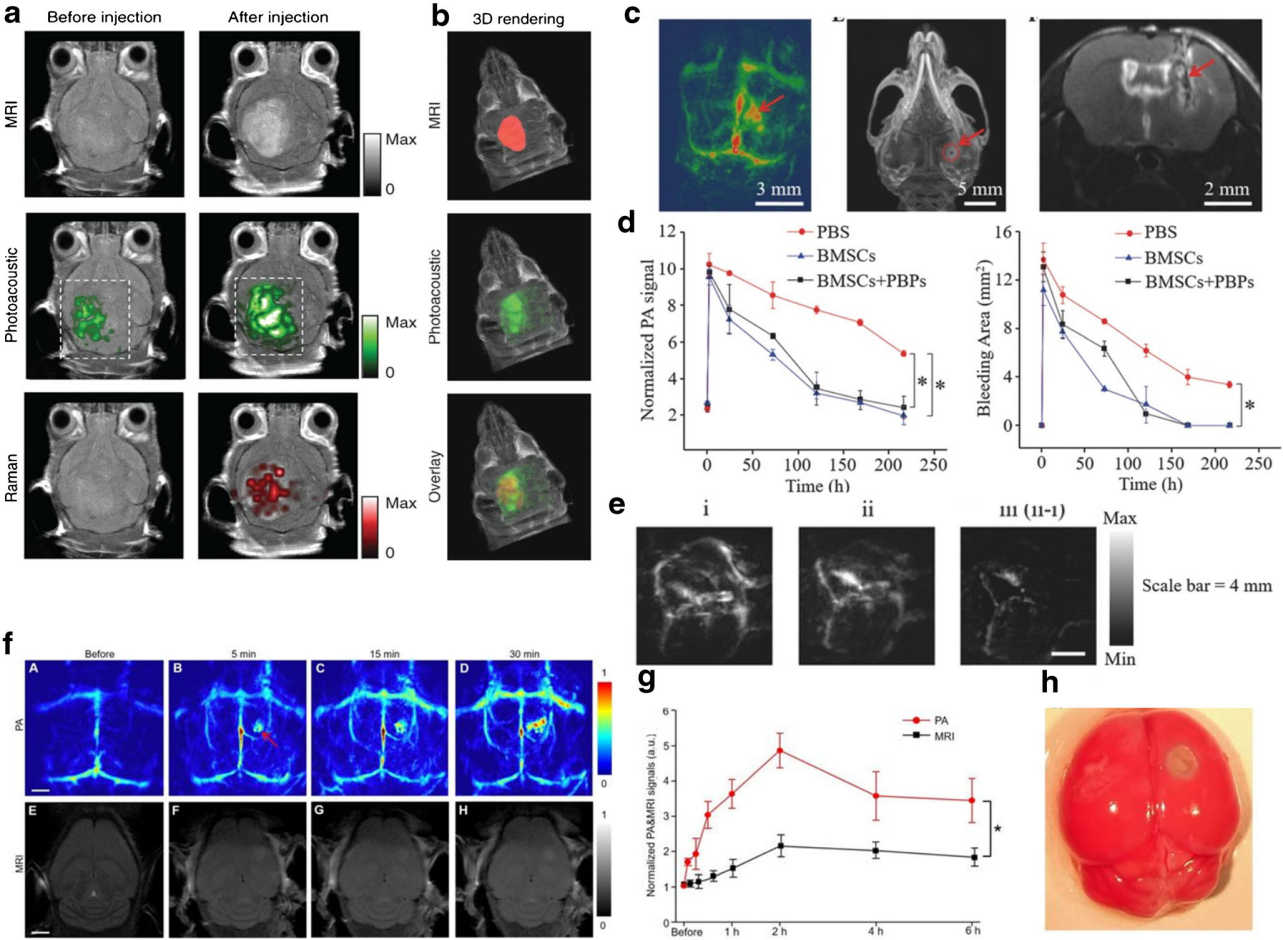
Multi-modality imaging studies. **a–b** Optoacoustic (OA), Raman, and MR images of the brain of orthotopic inoculation tumor-bearing mice before and after i.v. injection with multimodal probe. The post-injection images of all three modalities demonstrated clear tumor visualization. The OA and Raman images were co-registered with the MR image, demonstrating good co-localization between the three modalities. Adapted with permission from [84]; **c–e** stem cell imaging in brain injury model; **c** volumetric OA, CT, and MRI images of the mouse brain after cerebral injury. The wound hole marked by the red dotted line circle was induced by the steel needle; **d** normalized OA signal intensities of the damaged location at the predetermined time points. *P < 0.05; **e** OA images of the mouse brain (i) before and (ii) after a single injection of bone mesenchymal stem cells (BMSC) labeled with Prussian blue particles (PBPs). The image (iii) represents the delta image. Adapted with permission from [64]; **f–h** OA tomography (at 680 nm) and MRI of mouse brain in a photothrombosis stroke model at an early stage in vivo by Evan blue dye injection at varied time points upon injection; **g** normalized OA and MRI signals of mice brains in infarcted areas at varied time points upon Evan blue dye injection. *P < 0.05. **h** Triphenyl tetrazolium chloride staining in the brain of model mice. Adapted with permission from [144]

Kircher et al. (2012) developed a triple-modality MRI-OA-Raman nanoparticle for imaging of glioblastoma [84] (Fig. 6a–b). Liu et al. (2018) loaded molybdenum disulfide nanosheets with indocyanine green [104]. Glioblastoma could be clearly visualized after the probe injection. Song et al. (2019) developed a multi-modal probe for MRI and OA imaging for visualization of glioblastoma [105]. OA has been used to detect circulating breast cancer cells metastasizing to the brain [106]. After injecting gold nanoparticles directly into the tumor, the cells could be detected in the cerebrospinal fluid in the cisterna magna in the following days. Li et al. demonstrated tracking circulating tumor cells melanoma migration in the mouse brain in vivo using endogenous melanin signals by using tomographic OA imaging method termed SIP-PACT (Fig. 4h–k) [37]. Metastatic cells in the cerebrospinal fluid were detected before macroscopic brain metastases became apparent.

Various theranostic strategies have also been tested [36, 68, 87, 88, 90, 92, 93, 107, 108]. For example, Song et al. (2015) designed and synthesized a graphene oxide (rGO)-loaded ultra-small plasmonic gold nanorod vesicle [67]. The vesicle also exhibited a high loading capacity of doxorubicin; thus, it was selectively uptaken by the tumor following an intravenous injection where it was subsequently detected with OA. The absorption of light also led to local heating of tumor cells and release of the doxorubicin. The combination of photothermal and chemotherapeutic effects has led to reduction of the tumor volume. Guo et al. (2018) used a polymer nanoparticle that was conjugated with c-RGD peptide, and thus can target the α_v_β_3_ integrin receptor overexpressed on tumors [68].

Targeted particles were used to image gliomas with MSOT. Moreover, light in the second NIR window was used for phototherapy, which resulted in the inhibition of tumor growth and increased survival. Li et al. (2020) demonstrated the use of large amino acid mimicking quantum dots for imaging and treatment of glioma [109]. The functionalized quantum dots can bind to the large neutral amino acid transporter 1 that is overexpressed in most tumors, and are taken up by cells where they localize in the cytoplasm. The particles were localized after intravenous administration at the tumor site with MSOT in vivo. The quantum dots were also used as carriers for a number of DNA-damaging chemotherapy drugs. The group could show that the use of this particles as drug carriers enhanced delivery of drug to tumors and enhanced survival of tumor-bearing mice.

Tumor growth and tissue infiltration are associated with the activation of multiple pro-angiogenic signaling pathways. The newly formed blood vessels are highly irregular, have low perfusion and a partially or entirely compromised blood-brain barrier (BBB). Vascular leakage leads to edema and a high interstitial fluid pressure, thus preventing accumulation of therapeutic agents. Because of their high proliferation rate, tumor cells outgrow their vascular supply, causing intratumoral hypoxia, which increases tumor aggressiveness and resistance to radiation and chemotherapy. Thus, hemodynamic alterations may serve as a valuable imaging target. Burton et al. used MSOT to characterize glioblastoma hemodynamics in a mouse model [43]. The tumor displayed a strong Hb signal. Oxygenation measurements using a CO_2_ challenge also enable to localize the tumor mass. Furthermore, with injection of IntegriSense, the expression of the angiogenic α_v_β_3_ integrin receptor was directly visualized. Attia et al. (2016) showed with MSOT higher hemoglobin signal in the tumor periphery, reflecting the vascular heterogeneity of this tumor form with a higher vessel density at its boundary [110]. Moreover, by injection of the IRDye800CW 2-deoxyglucose, a higher glucose metabolism in the glioblastoma compared to the surrounding tissue was observed. Balasundaram et al. (2018) demonstrated the use of MSOT to retrieve information on tissue oxygenation for the purpose of cancer staging and monitoring of vascular-targeted treatment [111]. They showed in a glioblastoma model that the tumor has a hyperoxic and hypoxic phase during growth. Moreover, administration of combretastatin A4 phosphate, an agent that increases vascular permeability by increasing interstitial pressure reducing intratumoral blood flow, has resulted in measurable transient changes in tumor oxygenation.

Taken together, MSOT can be used to monitor a number of brain cancer hallmarks. Endogenous contrast is exploited to study tumor vascular function. Use of specific MSOT probes allows to detect tumor specific processes and use of particles as drug carriers can be used for theranostics.

#### Alzheimer’s disease

Alzheimer’s disease (AD) is the most common form of dementia and cognitive decline in the elderly [112]. As western societies are aging, the incidence is expected to increase dramatically. Despite major efforts to develop AD therapy, present medication can only retard the progress of AD and stabilize cognitive functions for a limited time, but there is no cure of the disease yet. Therapy needs to start at the earliest possible stage of AD, i.e., before cognitive decline is detectable, because neurodegenerative processes have already irreversibly damaged the brain. Robust, quantitative, and non-invasive biomarkers that identify the transition from normal aging to the onset of AD at an early presymptomatic stage are highly desired. Such biomarkers will enable an earlier onset of therapy to stabilize individuals at a higher cognitive level. In addition, such biomarkers will support the development of new treatments to cure the disease by enabling to act at a time when neurodegeneration can still be prevented, and by monitoring the effectiveness of a therapy. Its two major neuropathological features are the aggregation of fibrillar β-amyloid (Aβ) in amyloid deposits and neurofibrillary tangles of misfolded hyperphosphorylated tau protein [113]. Transgenic animal models that show abnormal Aβ and/or tau pathology have been developed and facilitated the mechanistic understanding of AD and therapeutic development [114]. A panel of optical imaging probes based on, e.g., Thioflavin T, Congo red derivatives, and luminescent conjugated oligothiophenes [100, 115–123] has been developed for imaging Aβ and tau targeting at the β-sheet fibril structure [124, 125]. Hu et al. were the first to show the use of OAM and Congo red in APP/PS1 mice to visualize Aβ plaques [126]. As Congo red does not cross the BBB, the probe had to be injected into the cisterna magna. Derivatives have been designed capable to cross the BBB thus providing an easy access to cerebral Aβ pathology with, e.g., croconium compounds (CDAs) by Liu et al. [100]. In vitro assays favored the use of CDA-3 for in vivo OA imaging of Aβ deposits. Indeed, after intravenous injection of CDA-3 into five familial AD and wild type animals, the probe was specifically retained in the brain of transgenic animals with specificity further demonstrated by stainings. Ni et al. applied oxazine derivative AOI987 [118, 127] in arcAβ and APP/PS1 mice and curcumin derivative CRANAD-2 in arcAβ mice (Fig. 5b) [119, 128], and demonstrated a higher cortical retention of Aβ deposits in transgenic models in vivo using MSOT. In addition to detecting the β-sheet of fibrils, many studies focus on targeting at copper ion which seems to be one of the main polyvalent metallic cations involved in Aβ plaque formation. Wang et al. have designed and synthesized an activatable probe RPS1 for MSOT imaging of brain copper accumulation [129], which can effectively cross the BBB. Upon chelation with Cu^2+^, the RPS1 probe becomes RPS1-Cu and generates strong OA signals. Tau imaging probes with suitable optical absorption spectrum such as PBB derivative could potentially be employed for MSOT imaging of tauopathy [130–132].

Alternative to the neuropathological pathway, neuroinflammation has been implicated to partake in the AD pathogenesis [133]. To study glial activation in AD, Park et al. (2019) have applied the probe CDnir7 to triple transgenic AD mice [134]. Specific retention of probe was observed with MSOT imaging in the cerebral cortex. Immunohistochemistry showed intracellular accumulation of probe in astrocytes and microglia/macrophage. Thus, the probe might enable to study the role of glial activations in the disease course.

Vascular pathways play important roles in AD [135]. The arcAβ model of amyloidosis exhibits strong vascular dysfunction [136–138]. Tissue oxygenation and CBF were respectively measured in arcAβ mice by means of MSOT and MRI [111]. Cerebral metabolic rate of oxygen was subsequently calculated based on these metrics, which was found significantly lower in aged arcAβ mice versus young mice and wild type controls. Therefore, MSOT allows to detect, quantify, and monitor Aβ deposition in AD, thus following the distribution and spread of the misfolded proteins across the brain, as well as to monitor response to therapies targeted against protein aggregation. In addition, mediators of other pathophysiological pathways can be visualized by OA.

#### Stroke

Stroke is the second highest cause of death globally and a leading cause of disability, with an increasing incidence in developing countries. The majority of ischemic stroke is caused by arterial occlusion. The acute reduction of CBF results in a shortage of glucose and oxygen to the supplied area. In the areas having the most severe reduction in CBF, termed the ischemic core, irreversible tissue damage occurs within minutes to hours after the onset of occlusion [139]. The surrounding tissue, referred to as ischemic penumbra, is still partially perfused although at a reduced rate. Management of stroke focuses on rapid restoration of CBF with intravenous thrombolysis and endovascular thrombectomy, which both reduce disability but are only effective and safe within a short and early time window.

With its central capability to monitor Hb and HbO_2_, MSOT has been used to study hemodynamic function and tissue oxygenation in rodent models of stroke. For instance, in a mouse model using intraluminal occlusion of the middle cerebral artery, Kneipp et al. (2014) have shown a reduction of CBV in the ischemic territory during the acute occlusion [140]. Furthermore, they demonstrated that area surrounding the core had an increased concentration of Hb, indicative of an increased oxygen extraction fraction in the penumbral area [141]. In the same mouse model, Ni et al. (2018) demonstrated a significant reduction of tissue oxygenation in the ischemic hemisphere and cortex during occlusion [142]. Tissue oxygenation was found to return to normal at 48 h after reperfusion. Vaas et al. (2017) showed that in the middle cerebral artery model, also, the external carotid artery is occluded, leading to a reduction in tissue oxygenation in extracerebral tissue with subsequent tissue damage by MSOT [143]. Work by Lv et al. (2020) used both the intraluminal model of the middle cerebral artery and a photothrombosis model of stroke to visualize reduction in tissue oxygenation in the ischemic territory within 6 h after onset of vessel occlusions using OAM [144]. In the case of photothrombosis, tissue oxygenation was followed for seven days, where eventually a gradual return to baseline oxygenation values in the tissue was observed. Work from Bandla et al. (2018) in the photothrombosis model showed that intravenous thrombolysis with recombinant tissue plasminogen activator leads to an improved perfusion of ischemic area and to a faster recovery of tissue oxygenation [145]. Sun et al. (2015) showed in a model of transient cerebral hypoxia-ischemia that the condition led to a severe reduction in tissue oxygenation, which persisted also after an experimental resolution of the condition [146]. The group further showed that treatment with the free radical scavenger Edaravone improved reoxygenation of tissue, demonstrating that reactive oxygen species are involved in the reperfusion and oxygenation deficit after stabling reperfusion. Secondary tissue damage occurs in the penumbra leading to growth of the core, where its molecular and cellular players have been identified in animal models of stroke. For example, Ni et al. (2018) have shown activity of matrix metalloproteinases using MSOT and an activatable probe at the subacute stage after middle cerebral artery occlusion and reperfusion [142]. BBB impairment is a consequence of matrix metalloproteinase activity [147]. Lv et al. (2020) showed extravasation of intravenously injected Evans blue, which binds to plasma albumin, after middle cerebral artery occlusion, indicative of BBB impairment [144, 148] (Fig. 6f–g). Thus, MSOT and other OA imaging methods are suitable for monitoring the hemodynamic and cellular processes during and following stroke.

#### Epilepsy

Epilepsy is the most common chronic brain disorder, affecting one percent of the population worldwide, spanning all age groups [149]. Epilepsy can substantially impair quality of life owing to seizures, comorbid mood and psychiatric disorders, and cognitive deficits. Seizures can be fatal owing to direct effects on autonomic and arousal functions or by leading to accidents. Although anti-epileptic drugs are available, about one-third of the patients with epilepsy remain refractory to current medical therapy. Surgery is an effective intervention for patients with pharmaco-resistant types of epilepsy. A prerequisite for complete surgical resection of the epileptogenic foci is the mapping of epilepsy networks. Epileptic seizures are clearly recognizable on EEG recordings as they are usually accompanied by bilateral 3 to 4 Hz spike–wave discharges. Hemodynamic changes are often used as surrogates for epileptic neuronal activity both in animal models and in the clinics [150]. Techniques such as intrinsic optical signal, fMRI, and SPECT are employed to assess these hemodynamic changes. Spiking neuronal activity generates a strong metabolic response that induce a focal increase in CBF and vessel dilation, with a measurable increase in local hemoglobin concentration [150]. OA is ideally suited to monitor hemodynamic changes in the brain and has been used to study different animal models of epilepsy. Measuring hemodynamic signals with high spatial and temporal resolution and wide spatial sampling together with the associated neuronal activity, e.g., by EEG during epileptic activity in the same preparation allows to understand the correlation between the spatial extent of perfusion, oxygenation, and neuronal activity.

The first application was demonstrated by Zhang et al. (2008) who studied focal seizures in the rat brain with OA. After microinjection of bicuculline, a gamma-aminobutyric acid (GABA)_A_ receptor antagonist, into the neocortex, the group observed increased OA contrast at the site of the seizure [151]. The work was later extended to monitor epileptic seizures with a real-time tomography imaging system in the same animal model [152]. The system allowed spatially and temporally following the seizure onset and spread. Further technical development allowed to monitor of focal epileptic activity with sub-second temporal resolution [153]. Following bicucullin administration in to the cortex, the authors noted not only a focal spot manifesting increased optical absorption contrast but also a decrease in signal surrounding the primary lesion and the appearance of homotopic foci in the contralateral cortex. Moreover, a network analysis was performed with the dynamic readings, which revealed significant changes in the whole-brain circuitry as a result of the epileptic activity. A similar system was used by Xiang et al. (2013) to monitor epileptic activity in real-time in the pentylenetetrazol (PTZ) model of generalized seizures in rats [154]. PTZ, a GABA_A_ receptor antagonist, was injected intraperitoneally and seizures were observed, with a clear correlation between interictal spike amplitude as recorded with EEG and OA signal. A MSOT system was used by Wang et al. (2014) to discriminate the response of Hb/HbO_2_ in the PTZ seizure rat model [155]. In their study, Wang et al. (2014) described a three-stage hemodynamic response in the superior sagittal sinus in relation to the EEG recordings of the seizure. During the first stage, there was a sudden decrease in HbO_2_ while Hb slightly increased as seizures initiated. In the second phase, as the seizures evolved, the HbO_2_ signal began to increase with a simultaneous increase in Hb. In the third phase, during resolution of the seizure activity, the Hb signal remained at diminished levels, while the hemoglobin increased.

Using a volumetric MSOT system and EEG, Gottschalk et al. (2017) studied the 4-aminopyridine (4-AP) model of focal seizures in mice [29]. Administration of 4-AP, a potassium channel blocker, has caused prolongation of action potentials and abnormal neural synchronization. At the epileptic foci, they observed an increase in oxygenated hemoglobin and decrease in Hb, concomitant with an increase in total hemoglobin levels with EEG activity. They also observed a delayed oxygenated hemoglobin signal in the thalamus, away from the epileptic focus correlated to the EEG activity [29]. The 4-AP mouse model was used by Zhang et al. (2018), who showed that the epileptic activity can generalize from the focal area across the whole brain [156]. In work by Rao et al. (2017), an approach to use dipicrylamine, a non-radiative voltage sensor, to monitor epileptic neuronal activity in conjunction with hemodynamic response in the 4-AP mouse model, was explored [157]. Upon injection of 4-AP, a strong voltage response signal change and a hemodynamic signal change were observed, where the voltage response signal changes were lower. The used voltage sensor works in the visible spectral range and thus is not suitable for sensing in deep brain tissue. Gottschalk et al. (2019) further showed a calcium signal increase with injection of PTZ in isolated GCaMP6f mouse brains, but not in control and phosphate-buffered saline injected brain [31] (Fig. 5c–f). Kang et al. (2019) proposed an alternative approach with the use of a near-infrared cyanine voltage-sensitive dye in the PTZ rat model of generalized seizures [158]. Administration of PTZ led to a measurable increase voltage response signal. Kang et al. (2020) further used the voltage-sensitive dye to monitor excitatory neuronal activity in the hippocampus evoked by infusion of *N*-methyl-d-aspartate (NMDA) in rat model [159]. Infusion of NMDA resulted in measurable increase in the voltage response signal.

Taken together, MSOT can be used to map epileptic seizures via both hemodynamic and neuronal activity. The technique may be well-suited to monitor the spread of activity across the brain in models of epilepsy and to monitor the effects of anti-epileptic drugs and interventions.

### Multimodal imaging

At present, fMRI based on BOLD signal is widely used for studying brain activity under resting state and stimulus-evoked conditions in humans and rodents [47, 48]. Simultaneous fluorescence Ca^2+^ imaging across the cortex and whole-brain fMRI at high field may serve to provide insights into the mechanisms of neurovascular coupling through the link between Ca^2+^ and BOLD signals [160]. Combining MSOT imaging approaches with other modalities having better soft tissue contrast, such as MRI or CT, has facilitated more accurate quantification [118, 161]. To this end, co-registration of images acquired by stand-alone MSOT and MRI scanners has been performed using MRI data as an anatomical reference for brain regional analysis [142] or by applying MRI/MSOT dual modality probes [86, 100, 162, 163]. The recent introduction of a hybrid scanner capable of concurrent MRI and volumetric MSOT recordings [164] holds great promise for improving the workflow, further complementing and validating functional and molecular readings by both modalities. All in all, various other multimodal combinations have been reported in the context of molecular imaging of brain diseases, including fluorescence/MSOT [107, 165], Raman/MSOT/MRI [84, 91], MSOT/MRI [64, 144], PET/MSOT [100], SPECT/MSOT [66], PET/MSOT [100], ultrasound/MSOT [166, 167], and fluorescence molecular tomography (FMT)/CT/MSOT [86] (Fig. 6).

### Outlook

At present, the real-time direct monitoring of large-scale neuronal activity can truly revolutionize brain research. Integrated neurophotonics has been introduced for dense volumetric imaging of brain circuit activity at deep brain has been proposed [168]. Hand-held MSOT technology was recently employed for the characterization of hemodynamic changes related with neuronal activity induced by optogenetic stimulation of intra-cortical connections in the rat brain [169]. MSOT imaging based on hemodynamic [32, 51] and calcium [31, 170] imaging has demonstrated the unique capacity to fill the gap between optical 2P microscopy [171, 172], and macroscopic hemodynamics and metabolism imaging using MRI [47, 48]. Furthermore, the use of optogenetics [173–175] and chemogenetic tools (DREADDs) [176] has enabled to modulate the activity of targeted neurons in animal models with high precision. Thus, combining MSOT imaging with optogenetics, chemogenetics and other neuromodulation techniques (e.g., ultrasound-based [50]) will allow for real-time whole-brain modulation and imaging pipeline, useful for elucidating the brain function and disease mechanisms.

### Future developments/challenges

OA is a rapidly evolving technique with outstanding challenges in regard to imaging methodologies and implementation of applicants still exists. Particular challenges are:

1. Technical issues in accurate signal quantification: a number of outstanding challenges exist for accurate quantification of MSOT data acquired from deep brain tissues. Those include the following: limited penetration depth; inability to accurately account for wavelength- and position-dependent light fluence distribution; image artifacts induced by skull aberrations and acoustic heterogeneities; and insufficient accuracy of the spectral unmixing algorithms for reconstructing chromophore concentration. This calls for development of more advanced algorithms for accurate modelling of the various experimental parameters, such as the excitation light fluence distribution [177], wavelength-dependent light attenuation [178], speed of sound distribution [179], frequency, and spatial response of the transducer arrays [180, 181], to name a few examples.
2. Artificial intelligence: different machine and deep learning algorithms have been exploited in OA imaging at different stages of the OA workflow, including data acquisition [182], image reconstruction [183], segmentation [184], artifact removal [183, 185], and spectral unmixing [186]. Rapid development of machine learning approaches for MSOT imaging can be foreseen as the vast amount of in vivo data is being accumulated across a variety of application fields.
3. Progress will also come from the design and synthesis of new specific OA probes for brain imaging. Probes should ideally have (1) suitable absorption spectrum in NIR or NIR II window that can be reliably unmixed from strong endogenous hemoglobin background, (2) BBB penetration, (3) low toxicity, (4) high sensitivity and specificity, (5) sufficient binding affinity, and (6) photostability.
4. Clinical translation: application of MSOT imaging in the clinical research such as dermatology and cancer has shown promising results in the recent years [187–190]. However, significant challenges exist for MSOT imaging of the human brain due to acoustic distortions introduced by the skull bone. Furthermore, to facilitate the clinical translation of MSOT imaging for precision diagnostics applications, there is an urgent need for standardized methodology, quality assurance, and consensus on data acquisition and analysis to enable accurate and reproducible comparisons of the performance across different MSOT imaging instruments and among different users [191, 192]. Addressing these outstanding challenges in the foreseeable future, OA will provide robust quantitative data, thus providing unique capabilities for in vivo interrogation of the structure and functions of the healthy and diseased brain.

## References

[1] Belliveau JW, Kennedy DN, McKinstry RC, Buchbinder BR, Weisskoff RM, Cohen MS (1991). Functional mapping of the human visual cortex by magnetic resonance imaging. Science..

[2] Lerch JP, van der Kouwe AJW, Raznahan A, Paus T, Johansen-Berg H, Miller KL (2017). Studying neuroanatomy using MRI. Nat Neurosci.

[3] Nordberg A, Rinne JO, Kadir A, Långström B (2010). The use of PET in Alzheimer disease. Nat Rev Neurol.

[4] Langen K-J, Galldiks N, Hattingen E, Shah NJ (2017). Advances in neuro-oncology imaging. Nat Rev Neurol.

[5] Macé E, Montaldo G, Cohen I, Baulac M, Fink M, Tanter M (2011). Functional ultrasound imaging of the brain. Nat Methods.

[6] Vahrmeijer AL, Hutteman M, van der Vorst JR, van de Velde CJH, Frangioni JV (2013). Image-guided cancer surgery using near-infrared fluorescence. Nat Rev Clin Oncol.

[7] Jack CR, Bennett DA, Blennow K, Carrillo MC, Dunn B, Haeberlein SB (2018). NIA-AA Research Framework: toward a biological definition of Alzheimer’s disease. Alzheimers Dement.

[8] Zijlmans M, Zweiphenning W, van Klink N (2019). Changing concepts in presurgical assessment for epilepsy surgery. Nat Rev Neurol.

[9] Bacskai BJ, Kajdasz ST, Christie RH, Carter C, Games D, Seubert P (2001). Imaging of amyloid-beta deposits in brains of living mice permits direct observation of clearance of plaques with immunotherapy. Nat Med.

[10] Keu KV, Witney TH, Yaghoubi S, Rosenberg J, Kurien A, Magnusson R, et al. Reporter gene imaging of targeted T cell immunotherapy in recurrent glioma. Sci Transl Med. 2017;9. 10.1126/scitranslmed.aag2196. 10.1126/scitranslmed.aag2196 PMC5260938 28100832

[11] Thomalla G, Simonsen CZ, Boutitie F, Andersen G, Berthezene Y, Cheng B (2018). MRI-guided thrombolysis for stroke with unknown time of onset. N Engl J Med.

[12] Klohs J, Rudin M (2011). Unveiling molecular events in the brain by noninvasive imaging. Neuroscientist..

[13] Zhong Y, Ma Z, Wang F, Wang X, Yang Y, Liu Y (2019). In vivo molecular imaging for immunotherapy using ultra-bright near-infrared-IIb rare-earth nanoparticles. Nat Biotechnol.

[14] Chen G, Cao Y, Tang Y, Yang X, Liu Y, Huang D (2020). Advanced near-infrared light for monitoring and modulating the spatiotemporal dynamics of cell functions in living systems. Adv Sci.

[15] Klohs J, Steinbrink J, Nierhaus T, Bourayou R, Lindauer U, Bahmani P (2006). Noninvasive near-infrared imaging of fluorochromes within the brain of live mice: an in vivo phantom study. Mol Imaging.

[16] Geraldes R, Ciccarelli O, Barkhof F, De Stefano N, Enzinger C, Filippi M (2018). The current role of MRI in differentiating multiple sclerosis from its imaging mimics. Nat Rev Neurol.

[17] Baron J-C (2018). Protecting the ischaemic penumbra as an adjunct to thrombectomy for acute stroke. Nat Rev Neurol.

[18] Deffieux T, Demene C, Pernot M, Tanter M (2018). Functional ultrasound neuroimaging: a review of the preclinical and clinical state of the art. Curr Opin Neurobiol.

[19] Lipsman N, Schwartz ML, Huang Y, Lee L, Sankar T, Chapman M (2013). MR-guided focused ultrasound thalamotomy for essential tremor: a proof-of-concept study. Lancet Neurol.

[20] Martínez-Fernández R, Rodríguez-Rojas R, Del Álamo M, Hernández-Fernández F, Pineda-Pardo JA, Dileone M (2018). Focused ultrasound subthalamotomy in patients with asymmetric Parkinso’s disease: a pilot study. Lancet Neurol.

[21] Tufail Y, Yoshihiro A, Pati S, Li MM, Tyler WJ (2011). Ultrasonic neuromodulation by brain stimulation with transcranial ultrasound. Nat Protoc.

[22] Wang LV, Yao J (2016). A practical guide to photoacoustic tomography in the life sciences. Nat Methods.

[23] Wang X, Pang Y, Ku G, Xie X, Stoica G, Wang LV (2003). Noninvasive laser-induced photoacoustic tomography for structural and functional in vivo imaging of the brain. Nat Biotechnol.

[24] Dima A, Burton NC, Ntziachristos V (2014). Multispectral optoacoustic tomography at 64, 128, and 256 channels. J Biomed Opt.

[25] Yao J, Wang L, Yang J-M, Maslov KI, Wong TTW, Li L (2015). High-speed label-free functional photoacoustic microscopy of mouse brain in action. Nat Methods.

[26] Gamelin J, Maurudis A, Aguirre A, Huang F, Guo P, Wang LV (2009). A real-time photoacoustic tomography system for small animals. Opt Express.

[27] Razansky D, Distel M, Vinegoni C, Ma R, Perrimon N, Köster RW (2009). Multispectral opto-acoustic tomography of deep-seated fluorescent proteins in vivo. Nat Photonics.

[28] Taruttis A, Morscher S, Burton NC, Razansky D, Ntziachristos V (2012). Fast multispectral optoacoustic tomography (MSOT) for dynamic imaging of pharmacokinetics and biodistribution in multiple organs. PLoS One.

[29] Gottschalk S, Fehm TF, Dean-Ben XL, Tsytsarev V, Razansky D (2017). Correlation between volumetric oxygenation responses and electrophysiology identifies deep thalamocortical activity during epileptic seizures. Neurophotonics..

[30] Deán-Ben XL, Razansky D (2014). Adding fifth dimension to optoacoustic imaging: volumetric time-resolved spectrally enriched tomography. Light: Sci & Appl.

[31] Gottschalk S, Degtyaruk O, Mc Larney B, Rebling J, Hutter MA, Deán-Ben XL (2019). Rapid volumetric optoacoustic imaging of neural dynamics across the mouse brain. Nat Biomed Eng.

[32] Gottschalk S, Fehm TF, Deán-Ben XL, Razansky D (2015). Noninvasive real-time visualization of multiple cerebral hemodynamic parameters in whole mouse brains using five-dimensional optoacoustic tomography. JCBFM..

[33] Cao R, Li J, Ning B, Sun N, Wang T, Zuo Z (2017). Functional and oxygen-metabolic photoacoustic microscopy of the awake mouse brain. NeuroImage..

[34] Ning B, Sun N, Cao R, Chen R, Kirk Shung K, Hossack JA (2015). Ultrasound-aided multi-parametric photoacoustic microscopy of the mouse brain. Sci Rep.

[35] Dean-Ben XL, Robin J, Ni R, Razansky D. Noninvasive three-dimensional optoacoustic localization microangiography of deep tissues. 2020. arXiv:2007.00372.

[36] Haedicke K, Agemy L, Omar M, Berezhnoi A, Roberts S, Longo-Machado C (2020). High-resolution optoacoustic imaging of tissue responses to vascular-targeted therapies. Nat Biomed Eng.

[37] Li L, Zhu L, Ma C, Lin L, Yao J, Wang L (2017). Single-impulse panoramic photoacoustic computed tomography of small-animal whole-body dynamics at high spatiotemporal resolution. Nat Biomed Eng.

[38] Kim J, Kim JY, Jeon S, Baik JW, Cho SH, Kim C (2019). Super-resolution localization photoacoustic microscopy using intrinsic red blood cells as contrast absorbers. Light: Sci & Appl.

[39] Wong TTW, Zhang R, Zhang C, Hsu H-C, Maslov KI, Wang L (2017). Label-free automated three-dimensional imaging of whole organs by microtomy-assisted photoacoustic microscopy. Nat Commun.

[40] Shi J, Wong TTW, He Y, Li L, Zhang R, Yung CS (2019). High-resolution, high-contrast mid-infrared imaging of fresh biological samples with ultraviolet-localized photoacoustic microscopy. Nat Photonics.

[41] Kisler K, Lazic D, Sweeney MD, Plunkett S, El Khatib M, Vinogradov SA (2018). In vivo imaging and analysis of cerebrovascular hemodynamic responses and tissue oxygenation in the mouse brain. Nat Protoc.

[42] Cao R, Li J, Kharel Y, Zhang C, Morris E, Santos WL (2018). Photoacoustic microscopy reveals the hemodynamic basis of sphingosine 1-phosphate-induced neuroprotection against ischemic stroke. Theranostics..

[43] Burton NC, Patel M, Morscher S, Driessen WH, Claussen J, Beziere N (2013). Multispectral opto-acoustic tomography (MSOT) of the brain and glioblastoma characterization. Neuroimage..

[44] Zhang P, Li L, Lin L, Shi J, Wang LV (2019). In vivo superresolution photoacoustic computed tomography by localization of single dyed droplets. Light: Sci & Appl.

[45] Kim C (2018). Beyond the acoustic diffraction limit: superresolution localization optoacoustic tomography (LOT). Light Sci Appl.

[46] Dean-Ben XL, Razansky D (2018). Localization optoacoustic tomography. Light: Sci & Appl.

[47] Schulz K, Sydekum E, Krueppel R, Engelbrecht CJ, Schlegel F, Schröter A (2012). Simultaneous BOLD fMRI and fiber-optic calcium recording in rat neocortex. Nat Methods.

[48] Schlegel F, Sych Y, Schroeter A, Stobart J, Weber B, Helmchen F (2018). Fiber-optic implant for simultaneous fluorescence-based calcium recordings and BOLD fMRI in mice. Nat Protoc.

[49] Olefir I, Ghazaryan A, Yang H, Malekzadeh-Najafabadi J, Glasl S, Symvoulidis P (2019). Spatial and spectral mapping and decomposition of neural dynamics and organization of the mouse brain with multispectral optoacoustic tomography. Cell Rep.

[50] Estrada H, Ozbek A, Robin J, Shoham S, Razansky D. Spherical array system for high precision transcranial ultrasound stimulation and optoacoustic imaging in rodents. IEEE Trans Ultrason Ferroelectr Freq Control. 2020;Pp. doi:10.1109/tuffc.2020.2994877. 10.1109/TUFFC.2020.2994877 PMC7952015 32406833

[51] Mc Larney B, Hutter MA, Degtyaruk O, Deán-Ben XL, Razansky D (2020). Monitoring of stimulus evoked murine somatosensory cortex hemodynamic activity with volumetric multi-spectral optoacoustic tomography. Front Neurosci.

[52] Deán-Ben XL, Gottschalk S, Mc Larney B, Shoham S, Razansky D (2017). Advanced optoacoustic methods for multiscale imaging of in vivo dynamics. Chem Soc Rev.

[53] Li Y, Li L, Zhu L, Maslov K, Shi J, Hu P (2020). Snapshot photoacoustic topography through an ergodic relay for high-throughput imaging of optical absorption. Nat Photonics.

[54] Ovsepian SV, Olefir I, Westmeyer G, Razansky D, Ntziachristos V (2017). Pushing the boundaries of neuroimaging with optoacoustics. Neuron..

[55] Weber J, Beard PC, Bohndiek SE (2016). Contrast agents for molecular photoacoustic imaging. Nat Methods.

[56] Pu K, Shuhendler AJ, Jokerst JV, Mei J, Gambhir SS, Bao Z (2014). Semiconducting polymer nanoparticles as photoacoustic molecular imaging probes in living mice. Nat Nanotechnol.

[57] Gujrati V, Mishra A, Ntziachristos V (2017). Molecular imaging probes for multi-spectral optoacoustic tomography. Chem Commun (Camb).

[58] De Luca M, Aiuti A, Cossu G, Parmar M, Pellegrini G, Robey PG (2019). Advances in stem cell research and therapeutic development. Nat Cell Biol.

[59] Chen PJ, Kang YD, Lin CH, Chen SY, Hsieh CH, Chen YY (2015). Multitheragnostic multi-GNRs crystal-seeded magnetic nanoseaurchin for enhanced in vivo mesenchymal-stem-cell homing, multimodal imaging, and stroke therapy. Adv Mater.

[60] Yin C, Wen G, Liu C, Yang B, Lin S, Huang J (2018). Organic semiconducting polymer nanoparticles for photoacoustic labeling and tracking of stem cells in the second near-infrared window. ACS Nano.

[61] Dhada KS, Hernandez DS, Suggs LJ (2019). In vivo photoacoustic tracking of mesenchymal stem cell viability. ACS Nano.

[62] Kim T, Lemaster JE, Chen F, Li J, Jokerst JV (2017). Photoacoustic imaging of human mesenchymal stem cells labeled with prussian blue-poly(l-lysine) nanocomplexes. ACS Nano.

[63] Kubelick KP, Snider EJ, Ethier CR, Emelianov S (2019). Development of a stem cell tracking platform for ophthalmic applications using ultrasound and photoacoustic imaging. Theranostics..

[64] Li W, Chen R, Lv J, Wang H, Liu Y, Peng Y (2018). In Vivo Photoacoustic imaging of brain injury and rehabilitation by high-efficient near-infrared dye labeled mesenchymal stem cells with enhanced brain barrier permeability. Adv Sci.

[65] Kang J, Kim D, Wang J, Han Y, Zuidema JM, Hariri A (2018). Enhanced performance of a molecular photoacoustic imaging agent by encapsulation in mesoporous silicon nanoparticles. Adv Mater.

[66] Yao M, Shi X, Zuo C, Ma M, Zhang L, Zhang H (2020). Engineering of SPECT/photoacoustic imaging/antioxidative stress triple-function nanoprobe for advanced mesenchymal stem cell therapy of cerebral ischemia. ACS Appl Mater Interfaces.

[67] Song J, Yang X, Jacobson O, Lin L, Huang P, Niu G (2015). Sequential drug release and enhanced photothermal and photoacoustic effect of hybrid reduced graphene oxide-loaded ultrasmall gold nanorod vesicles for cancer therapy. ACS Nano.

[68] Guo B, Sheng Z, Hu D, Liu C, Zheng H, Liu B (2018). Through scalp and skull NIR-II photothermal therapy of deep orthotopic brain tumors with precise photoacoustic imaging guidance. Adv Mater.

[69] Comenge J, Sharkey J, Fragueiro O, Wilm B, Brust M, Murray P (2018). Multimodal cell tracking from systemic administration to tumour growth by combining gold nanorods and reporter genes. Elife..

[70] Qian Y, Piatkevich KD, Mc Larney B, Abdelfattah AS, Mehta S, Murdock MH (2019). A genetically encoded near-infrared fluorescent calcium ion indicator. Nat Methods.

[71] Mishra K, Stankevych M, Fuenzalida-Werner JP, Grassmann S, Gujrati V, Huang Y (2020). Multiplexed whole-animal imaging with reversibly switchable optoacoustic proteins. Sci Adv.

[72] Yao J, Kaberniuk AA, Li L, Shcherbakova DM, Zhang R, Wang L (2016). Multiscale photoacoustic tomography using reversibly switchable bacterial phytochrome as a near-infrared photochromic probe. Nat Methods.

[73] Li L, Shemetov AA, Baloban M, Hu P, Zhu L, Shcherbakova DM (2018). Small near-infrared photochromic protein for photoacoustic multi-contrast imaging and detection of protein interactions in vivo. Nat Commun.

[74] Cai Z, Zhu L, Wang M, Roe AW, Xi W, Qian J (2020). NIR-II fluorescence microscopic imaging of cortical vasculature in non-human primates. Theranostics..

[75] Zhang XD, Wang H, Antaris AL, Li L, Diao S, Ma R (2016). Traumatic brain injury imaging in the second near-infrared window with a molecular fluorophore. Adv Mater.

[76] Roberts S, Seeger M, Jiang Y, Mishra A, Sigmund F, Stelzl A (2018). Calcium sensor for photoacoustic imaging. J Am Chem Soc.

[77] Fosque BF, Sun Y, Dana H, Yang CT, Ohyama T, Tadross MR (2015). Neural circuits. Labeling of active neural circuits in vivo with designed calcium integrators. Science..

[78] Shemetov AA, Monakhov MV, Zhang Q, Canton-Josh JE, Kumar M, Chen M, et al. A near-infrared genetically encoded calcium indicator for in vivo imaging. Nat Biotechnol. 2020. 10.1038/s41587-020-0710-1. 10.1038/s41587-020-0710-1 PMC7956128 33106681

[79] Stobart JL, Ferrari KD, Barrett MJP, Glück C, Stobart MJ, Zuend M (2018). Cortical circuit activity evokes rapid astrocyte calcium signals on a similar timescale to neurons. Neuron.

[80] Monakhov MV, Matlashov ME, Colavita M, Song C, Shcherbakova DM, Antic SD (2020). Screening and cellular characterization of genetically encoded voltage indicators based on near-infrared fluorescent proteins. ACS Chem Neurosci.

[81] Bindocci E, Savtchouk I, Liaudet N, Becker D, Carriero G, Volterra A. Three-dimensional Ca2+ imaging advances understanding of astrocyte biology. Science. 2017, 356:eaai8185. 10.1126/science.aai8185. 10.1126/science.aai8185 28522470

[82] Dean-Ben XL, Sela G, Lauri A, Kneipp M, Ntziachristos V, Westmeyer GG (2016). Functional optoacoustic neuro-tomography for scalable whole-brain monitoring of calcium indicators. Light Sci Appl.

[83] Yankeelov TE, Abramson RG, Quarles CC (2014). Quantitative multimodality imaging in cancer research and therapy. Nat Rev Clin Oncol.

[84] Kircher MF, de la Zerda A, Jokerst JV, Zavaleta CL, Kempen PJ, Mittra E (2012). A brain tumor molecular imaging strategy using a new triple-modality MRI-photoacoustic-Raman nanoparticle. Nat Med.

[85] Ouyang J, Sun L, Zeng Z, Zeng C, Zeng F, Wu S (2020). Nanoaggregate probe for breast cancer metastasis through multispectral optoacoustic tomography and aggregation-induced NIR-I/II fluorescence imaging. Angew Chem Int Ed Eng.

[86] Huang W, Wang K, An Y, Meng H, Gao Y, Xiong Z (2020). In vivo three-dimensional evaluation of tumour hypoxia in nasopharyngeal carcinomas using FMT-CT and MSOT. Eur J Nucl Med Mol Imaging.

[87] Chen Q, Liang C, Sun X, Chen J, Yang Z, Zhao H (2017). H2O2-responsive liposomal nanoprobe for photoacoustic inflammation imaging and tumor theranostics via in vivo chromogenic assay. Proc Natl Acad Sci.

[88] Guo B, Chen J, Chen N, Middha E, Xu S, Pan Y (2019). High-resolution 3D NIR-II photoacoustic imaging of cerebral and tumor vasculatures using conjugated polymer nanoparticles as contrast agent. Adv Mater.

[89] Thawani JP, Amirshaghaghi A, Yan L, Stein JM, Liu J, Tsourkas A. Photoacoustic-guided surgery with indocyanine green-coated superparamagnetic iron oxide nanoparticle clusters. Small. 2017;13. 10.1002/smll.201701300. 10.1002/smll.201701300 PMC5884067 28748623

[90] Duan Y, Hu D, Guo B, Shi Q, Wu M, Xu S (2020). Nanostructural control enables optimized photoacoustic–fluorescence–magnetic resonance multimodal imaging and photothermal therapy of brain tumor. Adv Funct Mater.

[91] Neuschmelting V, Harmsen S, Beziere N, Lockau H, Hsu HT, Huang R (2018). Dual-modality surface-enhanced resonance Raman scattering and multispectral optoacoustic tomography nanoparticle approach for brain tumor delineation. Small..

[92] Yang Z, Du Y, Sun Q, Peng Y, Wang R, Zhou Y (2020). Albumin-based nanotheranostic probe with hypoxia alleviating potentiates synchronous multimodal imaging and phototherapy for glioma. ACS Nano.

[93] You Q, Zhang K, Liu J, Liu C, Wang H, Wang M (2020). Persistent regulation of tumor hypoxia microenvironment via a bioinspired PT-based oxygen nanogenerator for multimodal imaging-guided synergistic phototherapy. Adv Sci.

[94] Bao X, Yuan Y, Chen J, Zhang B, Li D, Zhou D (2018). In vivo theranostics with near-infrared-emitting carbon dots—highly efficient photothermal therapy based on passive targeting after intravenous administration. Light: Sci & Appl.

[95] Shashkov EV, Everts M, Galanzha EI, Zharov VP (2008). Quantum dots as multimodal photoacoustic and photothermal contrast agents. Nano Lett.

[96] Song G, Kenney M, Chen Y-S, Zheng X, Deng Y, Chen Z (2020). Carbon-coated FeCo nanoparticles as sensitive magnetic-particle-imaging tracers with photothermal and magnetothermal properties. Nat Biomed Eng.

[97] Xie H, Liu M, You B, Luo G, Chen Y, Liu B (2020). Biodegradable Bi2O2Se quantum dots for photoacoustic imaging-guided cancer photothermal therapy. Small..

[98] Zhan C, Huang Y, Lin G, Huang S, Zeng F, Wu S (2019). A gold nanocage/cluster hybrid structure for whole-body multispectral optoacoustic tomography imaging, EGFR inhibitor delivery, and photothermal therapy. Small..

[99] Zhou Z, Li B, Shen C, Wu D, Fan H, Zhao J, et al. Metallic 1 T phase enabling MoS(2) nanodots as an efficient agent for photoacoustic imaging guided photothermal therapy in the near-infrared-II window. Small. 2020:e2004173. 10.1002/smll.202004173. 10.1002/smll.202004173 33006243

[100] Ke K, Yang W, Xie X, Liu R, Wang LL, Lin WW (2017). Copper manganese sulfide nanoplates: a new two-dimensional theranostic nanoplatform for MRI/MSOT dual-modal imaging-guided photothermal therapy in the second near-infrared window. Theranostics..

[101] Takakusagi Y, Naz S, Takakusagi K, Ishima M, Murata H, Ohta K (2018). A multimodal molecular imaging study evaluates pharmacological alteration of the tumor microenvironment to improve radiation response. Cancer Res.

[102] Knauth M, Aras N, Wirtz CR, Dörfler A, Engelhorn T, Sartor K (1999). Surgically induced intracranial contrast enhancement: potential source of diagnostic error in intraoperative MR imaging. AJNR Am J Neuroradiol.

[103] Deliolanis NC, Ale A, Morscher S, Burton NC, Schaefer K, Radrich K (2014). Deep-tissue reporter-gene imaging with fluorescence and optoacoustic tomography: a performance overview. Mol Imaging Biol.

[104] Liu C, Chen J, Zhu Y, Gong X, Zheng R, Chen N (2018). Highly sensitive MoS(2)-indocyanine green hybrid for photoacoustic imaging of orthotopic brain glioma at deep site. Nano Lett.

[105] Song G, Zheng X, Wang Y, Xia X, Chu S, Rao J (2019). A magneto-optical nanoplatform for multimodality imaging of tumors in mice. ACS Nano.

[106] Nedosekin DA, Juratli MA, Sarimollaoglu M, Moore CL, Rusch NJ, Smeltzer MS (2013). Photoacoustic and photothermal detection of circulating tumor cells, bacteria and nanoparticles in cerebrospinal fluid in vivo and ex vivo. J Biophotonics.

[107] Guo B, Feng Z, Hu D, Xu S, Middha E, Pan Y (2019). Precise deciphering of brain vasculatures and microscopic tumors with dual NIR-II fluorescence and photoacoustic imaging. Adv Mater.

[108] Hai P, Imai T, Xu S, Zhang R, Aft RL, Zou J (2019). High-throughput, label-free, single-cell photoacoustic microscopy of intratumoral metabolic heterogeneity. Nat Biomed Eng.

[109] Li S, Su W, Wu H, Yuan T, Yuan C, Liu J (2020). Targeted tumour theranostics in mice via carbon quantum dots structurally mimicking large amino acids. Nat Biomed Eng.

[110] Attia AB, Ho CJ, Chandrasekharan P, Balasundaram G, Tay HC, Burton NC (2016). Multispectral optoacoustic and MRI coregistration for molecular imaging of orthotopic model of human glioblastoma. J Biophotonics.

[111] Balasundaram G, Ding L, Li X, Attia ABE, Dean-Ben XL, Ho CJH (2018). Noninvasive anatomical and functional imaging of orthotopic glioblastoma development and therapy using multispectral optoacoustic tomography. Transl Oncol.

[112] Boyle PA, Yu L, Leurgans SE, Wilson RS, Brookmeyer R, Schneider JA (2019). Attributable risk of Alzheimer’s dementia attributed to age-related neuropathologies. Ann Neurol.

[113] Braak H, Braak E. Neuropathological stageing of Alzheimer-related changes. Acta Neuropathol. 1991;82. 10.1007/bf00308809. 10.1007/BF00308809 1759558

[114] Klohs J, Rudin M, Shimshek DR, Beckmann N (2014). Imaging of cerebrovascular pathology in animal models of Alzheimer’s disease. Front Aging Neurosci.

[115] Ono M, Sahara N, Kumata K, Ji B, Ni R, Koga S (2017). Distinct binding of PET ligands PBB3 and AV-1451 to tau fibril strains in neurodegenerative tauopathies. Brain..

[116] Zhou J, Jangili P, Son S, Ji MS, Won M, Kim JS (2020). Fluorescent diagnostic probes in neurodegenerative diseases. Adv Mater.

[117] Ni R, Gillberg PG, Bogdanovic N, Viitanen M, Myllykangas L, Nennesmo I (2017). Amyloid tracers binding sites in autosomal dominant and sporadic Alzheimer’s disease. Alzheimers Dement.

[118] Ni R, Dean-Ben XL, Kirschenbaum D, Rudin M, Chen Z, Crimi A, et al. Whole brain optoacoustic tomography reveals strain-specific regional beta-amyloid densities in Alzheimer’s disease amyloidosis models. bioRxiv. 2020:DOI: 10.1101/2020.02.25.964064.

[119] Ni R, Villois A, Dean-Ben XL, Chen Z, Vaas M, Stavrakis S (2020). In-vitro and in-vivo characterization of CRANAD-2 for multi-spectral optoacoustic tomography and fluorescence imaging of amyloid-beta deposits in Alzheimer mice. bioRxiv.

[120] Ni R, Chen Z, Shi G, Villois A, Zhou Q, Arosio P, et al. Transcranial in vivo detection of amyloid-beta at single plaque resolution with large-field multifocal illumination fluorescence microscopy. bioRxiv. 2020:2020.02.01.929844. doi:10.1101/2020.02.01.929844.

[121] Shirani H, Linares M, Sigurdson CJ, Lindgren M, Norman P, Nilsson KPR (2015). A palette of fluorescent thiophene-based ligands for the identification of protein aggregates. Chemistry..

[122] Ni R, Chen Z, Gerez JA, Shi G, Zhou Q, Riek R (2020). Detection of cerebral tauopathy in P301L mice using high-resolution large-field multifocal illumination fluorescence microscopy. Biomed Opt Express.

[123] Ni R, Gillberg PG, Bergfors A, Marutle A, Nordberg A (2013). Amyloid tracers detect multiple binding sites in Alzheimer’s disease brain tissue. Brain..

[124] Rodriguez-Vieitez E, Ni R, Gulyas B, Toth M, Haggkvist J, Halldin C (2015). Astrocytosis precedes amyloid plaque deposition in Alzheimer APPswe transgenic mouse brain: a correlative positron emission tomography and in vitro imaging study. Eur J Nucl Med Mol Imaging.

[125] Ishikawa A, Tokunaga M, Maeda J, Minamihisamatsu T, Shimojo M, Takuwa H (2018). In vivo visualization of tau accumulation, microglial activation, and brain atrophy in a mouse model of tauopathy rTg4510. J Alzheimers Dis.

[126] Hu S, Yan P, Maslov K, Lee JM, Wang LV (2009). Intravital imaging of amyloid plaques in a transgenic mouse model using optical-resolution photoacoustic microscopy. Opt Lett.

[127] Hintersteiner M, Enz A, Frey P, Jaton AL, Kinzy W, Kneuer R (2005). In vivo detection of amyloid-beta deposits by near-infrared imaging using an oxazine-derivative probe. Nat Biotechnol.

[128] Ran C, Xu X, Raymond SB, Ferrara BJ, Neal K, Bacskai BJ (2009). Design, synthesis, and testing of difluoroboron-derivatized curcumins as near-infrared probes for in vivo detection of amyloid-β deposits. J Am Chem Soc.

[129] Wang S, Sheng Z, Yang Z, Hu D, Long X, Feng G (2019). Activatable small-molecule photoacoustic probes that cross the blood-brain barrier for visualization of copper(II) in mice with Alzheimer’s disease. Angew Chem Int Ed Eng.

[130] Maruyama M, Shimada H, Suhara T, Shinotoh H, Ji B, Maeda J (2013). Imaging of tau pathology in a tauopathy mouse model and in Alzheimer patients compared to normal controls. Neuron.

[131] Okamura N, Furumoto S, Fodero-Tavoletti MT, Mulligan RS, Harada R, Yates P (2014). Non-invasive assessment of Alzheimer’s disease neurofibrillary pathology using 18F-THK5105 PET. Brain..

[132] Verwilst P, Kim HS, Kim S, Kang C, Kim JS (2018). Shedding light on tau protein aggregation: the progress in developing highly selective fluorophores. Chem Soc Rev.

[133] Heneka MT, Carson MJ, El Khoury J, Landreth GE, Brosseron F, Feinstein DL (2015). Neuroinflammation in Alzheimer’s disease. Lancet Neurol.

[134] Park S-J, Ho CJH, Arai S, Samanta A, Olivo M, Chang Y-T (2019). Visualizing Alzheimer’s disease mouse brain with multispectral optoacoustic tomography using a fluorescent probe, CDnir7. Sci Rep.

[135] Klohs J. An integrated view on vascular dysfunction in Alzheimer’s disease. Neurodegener Dis. 2020. 10.1159/000505625. 10.1159/000505625 32062666

[136] Klohs J, Deistung A, Ielacqua G, Seuwen A, Kindler D, Schweser F (2016). Quantitative assessment of microvasculopathy in arcAβ mice with USPIO-enhanced gradient echo MRI. J Cereb Blood Flow Metab.

[137] Ielacqua GDSF, Füchtemeier M, Xandry J, Rudin M, Klohs J (2016). Magnetic resonance q mapping reveals a decrease in microvessel density in the arcAβ mouse model of cerebral amyloidosis. Front Aging Neurosci.

[138] Ni R, Kindler DR, Waag R, Rouault M, Ravikumar P, Nitsch R (2019). fMRI reveals mitigation of cerebrovascular dysfunction by bradykinin receptors 1 and 2 inhibitor noscapine in a mouse model of cerebral amyloidosis. Front Aging Neurosci.

[139] Moskowitz MA, Lo EH, Iadecola C (2010). The science of stroke: mechanisms in search of treatments. Neuron..

[140] Kneipp M, Turner J, Hambauer S, Krieg SM, Lehmberg J, Lindauer U (2014). Functional real-time optoacoustic imaging of middle cerebral artery occlusion in mice. PLoS One.

[141] Luo Y, Gong Z, Zhou Y, Chang B, Chai C, Liu T (2017). Increased susceptibility of asymmetrically prominent cortical veins correlates with misery perfusion in patients with occlusion of the middle cerebral artery. Eur Radiol.

[142] Ni R, Vaas M, Ren W, Klohs J (2018). Non-invasive detection of acute cerebral hypoxia and subsequent matrix-metalloproteinase activity in a mouse model of cerebral ischemia using multispectral-optoacoustic-tomography. Neurophotonics..

[143] Vaas M, Ni R, Rudin M, Kipar A, Klohs J (2017). Extracerebral tissue damage in the intraluminal filament mouse model of middle cerebral artery occlusion. Front Neurol.

[144] Lv J, Li S, Zhang J, Duan F, Wu Z, Chen R (2020). In vivo photoacoustic imaging dynamically monitors the structural and functional changes of ischemic stroke at a very early stage. Theranostics..

[145] Bandla A, Liao LD, Chan SJ, Ling JM, Liu YH, Shih YI (2018). Simultaneous functional photoacoustic microscopy and electrocorticography reveal the impact of rtPA on dynamic neurovascular functions after cerebral ischemia. J Cereb Blood Flow Metab.

[146] Sun YY, Li Y, Wali B, Li Y, Lee J, Heinmiller A (2015). Prophylactic edaravone prevents transient hypoxic-ischemic brain injury: implications for perioperative neuroprotection. Stroke..

[147] Rosenberg GA, Estrada EY, Dencoff JE (1998). Matrix metalloproteinases and TIMPs are associated with blood-brain barrier opening after reperfusion in rat brain. Stroke..

[148] Klohs J, Baeva N, Steinbrink J, Bourayou R, Boettcher C, Royl G (2009). In vivo near-infrared fluorescence imaging of matrix metalloproteinase activity after cerebral ischemia. J Cereb Blood Flow Metab.

[149] Devinsky O, Vezzani A, O'Brien TJ, Jette N, Scheffer IE, de Curtis M (2018). Epilepsy. Nat Rev Dis Primers.

[150] Ma H, Zhao M, Suh M, Schwartz TH (2009). Hemodynamic surrogates for excitatory membrane potential change during interictal epileptiform events in rat neocortex. J Neurophysiol.

[151] Zhang Q, Liu Z, Carney PR, Yuan Z, Chen H, Roper SN (2008). Non-invasive imaging of epileptic seizures in vivo using photoacoustic tomography. Phys Med Biol.

[152] Wang B, Xiang L, Jiang MS, Yang J, Zhang Q, Carney PR (2012). Photoacoustic tomography system for noninvasive real-time three-dimensional imaging of epilepsy. Biomed Opt Expr.

[153] Xiang L, Ji L, Zhang T, Wang B, Yang J, Zhang Q (2013). Noninvasive real time tomographic imaging of epileptic foci and networks. NeuroImage..

[154] Xiang L, Wang B, Ji L, Jiang H (2013). 4-D photoacoustic tomography. Sci Rep.

[155] Wang B, Xiao J, Jiang H (2014). Simultaneous real-time 3D photoacoustic tomography and EEG for neurovascular coupling study in an animal model of epilepsy. J Neural Eng.

[156] Zhang P, Li L, Lin L, Hu P, Shi J, He Y (2018). High-resolution deep functional imaging of the whole mouse brain by photoacoustic computed tomography in vivo. J Biophotonics.

[157] Rao B, Zhang R, Li L, Shao J-Y, Wang LV (2017). Photoacoustic imaging of voltage responses beyond the optical diffusion limit. Sc Rep.

[158] Kang J, Zhang HK, Kadam SD, Fedorko J, Valentine H, Malla AP, et al. Transcranial recording of electrophysiological neural activity in the rodent brain in vivo using functional photoacoustic imaging of near-infrared voltage-sensitive dye. Front Neurosci. 2019;13. 10.3389/fnins.2019.00579. 10.3389/fnins.2019.00579 PMC6696882 31447622

[159] Kang J, Kadam SD, Elmore JS, Sullivan BJ, Valentine H, Malla AP (2020). Transcranial photoacoustic imaging of NMDA-evoked focal circuit dynamics in the rat hippocampus. J Neural Eng.

[160] Lake EMR, Ge X, Shen X, Herman P, Hyder F, Cardin JA, et al. Simultaneous cortex-wide fluorescence Ca2+ imaging and whole-brain fMRI. Nat Methods. 2020. 10.1038/s41592-020-00984-6. 10.1038/s41592-020-00984-6 PMC7704940 33139894

[161] Ren W, Skulason H, Schlegel F, Rudin M, Klohs J, Ni R (2019). Automated registration of magnetic resonance imaging and optoacoustic tomography data for experimental studies. Neurophotonics..

[162] Bouchard L-S, Anwar MS, Liu GL, Hann B, Xie ZH, Gray JW (2009). Picomolar sensitivity MRI and photoacoustic imaging of cobalt nanoparticles. Proc Natl Acad Sci.

[163] Wang S, You Q, Wang J, Song Y, Cheng Y, Wang Y (2019). MSOT/CT/MR imaging-guided and hypoxia-maneuvered oxygen self-supply radiotherapy based on one-pot MnO(2)-mSiO(2)@Au nanoparticles. Nanoscale..

[164] Ren W, Deán-Ben XL, Augath M-A, Razansky D (2020). Development of concurrent magnetic resonance imaging and volumetric optoacoustic tomography: a phantom feasibility study. J Biophotonics.

[165] Chen Z, Mu X, Han Z, Yang S, Zhang C, Guo Z (2019). An optical/photoacoustic dual-modality probe: ratiometric in/ex vivo imaging for stimulated H2S upregulation in mice. J Am Chem Soc.

[166] Chen H, Huang Y, Li B, Liao W, Zhang G, Lin Z (2015). Efficient orthogonally polarized dual-wavelength Nd:LaMgB5O10 laser. Opt Lett.

[167] Ivan O, Elena M, Neal CB, Saak VO, Vasilis N (2016). Hybrid multispectral optoacoustic and ultrasound tomography for morphological and physiological brain imaging. J Biomed Opt.

[168] Moreaux LC, Yatsenko D, Sacher WD, Choi J, Lee C, Kubat NJ (2020). Integrated neurophotonics: toward dense volumetric interrogation of brain circuit activity—at depth and in real time. Neuron..

[169] Ovsepian SV, Jiang Y, Sardella TCP, Malekzadeh-Najafabadi J, Burton NC, Yu X (2020). Visualizing cortical response to optogenetic stimulation and sensory inputs using multispectral handheld optoacoustic imaging. Photoacoustics..

[170] Degtyaruk O, Mc Larney B, Deán-Ben X, Shoham S, Razansky D (2019). Optoacoustic calcium imaging of deep brain activity in an intracardially perfused mouse brain model. Photonics..

[171] Helmchen F, Denk W (2005). Deep tissue two-photon microscopy. Nat Methods.

[172] Kleinfeld D, Mitra PP, Helmchen F, Denk W (1998). Fluctuations and stimulus-induced changes in blood flow observed in individual capillaries in layers 2 through 4 of rat neocortex. Proc Natl Acad Sci.

[173] Adam Y, Kim JJ, Lou S, Zhao Y, Xie ME, Brinks D (2019). Voltage imaging and optogenetics reveal behaviour-dependent changes in hippocampal dynamics. Nature..

[174] Kim TI, McCall JG, Jung YH, Huang X, Siuda ER, Li Y (2013). Injectable, cellular-scale optoelectronics with applications for wireless optogenetics. Science..

[175] Deisseroth K (2011). Optogenetics. Nat Methods.

[176] Roth BL (2016). DREADDs for Neuroscientists. Neuron..

[177] Brochu FM, Brunker J, Joseph J, Tomaszewski MR, Morscher S, Bohndiek SE (2017). Towards quantitative evaluation of tissue absorption coefficients using light fluence correction in optoacoustic tomography. IEEE Trans Med Imaging.

[178] Tzoumas S, Nunes A, Olefir I, Stangl S, Symvoulidis P, Glasl S (2016). Eigenspectra optoacoustic tomography achieves quantitative blood oxygenation imaging deep in tissues. Nat Commun.

[179] Guo X, Ding Y, Duan Y, Ni X (2019). Nonreciprocal metasurface with space–time phase modulation. Light: Sci & Appl.

[180] Ding L, Dean-Ben XL, Burton NC, Sobol RW, Ntziachristos V, Razansky D (2017). Constrained inversion and spectral unmixing in multispectral optoacoustic tomography. IEEE Trans Med Imaging.

[181] Rosenthal A, Ntziachristos V, Razansky D (2011). Optoacoustic methods for frequency calibration of ultrasonic sensors. IEEE Trans Ultrason Ferroelectr Freq Control.

[182] Davoudi N, Deán-Ben XL, Razansky D (2019). Deep learning optoacoustic tomography with sparse data. Nat Machine Intelligence.

[183] Lan H, Jiang D, Yang C, Gao F, Gao F (2020). Y-Net: Hybrid deep learning image reconstruction for photoacoustic tomography in vivo. Photoacoustics..

[184] Lafci B, Mercep E, Morscher S, Dean-Ben XL, Razansky D. Deep learning for automatic segmentation of hybrid optoacoustic ultrasound (OPUS) images. IEEE Trans Ultrason Ferroelectr Freq Control. 2020. 10.1109/tuffc.2020.3022324. 10.1109/TUFFC.2020.3022324 32894712

[185] Allman D, Reiter A, Bell MAL (2018). Photoacoustic source detection and reflection artifact removal enabled by deep learning. IEEE Trans Med Imaging.

[186] Olefir I, Tzoumas S, Restivo C, Mohajerani P, Xing L, Ntziachristos V. Deep learning based spectral unmixing for optoacoustic imaging of tissue oxygen saturation. IEEE Trans Med Imaging. 2020. doi:10.1109/tmi.2020.3001750. 10.1109/TMI.2020.3001750 PMC7671861 32746111

[187] Masthoff M, Helfen A, Claussen J, Karlas A, Markwardt NA, Ntziachristos V (2018). Use of multispectral optoacoustic tomography to diagnose vascular malformations. JAMA Dermatol.

[188] Knieling F, Neufert C, Hartmann A, Claussen J, Urich A, Egger C (2017). Multispectral optoacoustic tomography for assessment of Crohn’s disease activity. N Engl J Med.

[189] Deán-Ben XL, Razansky D (2013). Portable spherical array probe for volumetric real-time optoacoustic imaging at centimeter-scale depths. Opt Express.

[190] Ivankovic I, Merčep E, Schmedt CG, Deán-Ben XL, Razansky D (2019). Real-time volumetric assessment of the human carotid artery: handheld multispectral optoacoustic tomography. Radiology..

[191] Waterhouse DJ, Fitzpatrick CRM, Pogue BW, O’Connor JPB, Bohndiek SE (2019). A roadmap for the clinical implementation of optical-imaging biomarkers. Nat Biomed Eng.

[192] Joseph J, Tomaszewski MR, Quiros-Gonzalez I, Weber J, Brunker J, Bohndiek SE (2017). Evaluation of precision in optoacoustic tomography for preclinical imaging in living subjects. J Nucl Med.

[193] Nasiriavanaki M, Xia J, Wan H, Bauer AQ, Culver JP, Wang LV (2014). High-resolution photoacoustic tomography of resting-state functional connectivity in the mouse brain. Proc Natl Acad Sci.

[194] Matthews TP, Zhang C, Yao D-K, Maslov K, Wang LV (2014). Label-free photoacoustic microscopy of peripheral nerves. J Biomed Opt.

[195] Wu W, Wang P, Cheng JX, Xu XM (2014). Assessment of white matter loss using bond-selective photoacoustic imaging in a rat model of contusive spinal cord injury. J Neurotrauma.

[196] Changalvaie B, Han S, Moaseri E, Scaletti F, Truong L, Caplan R (2019). Indocyanine green J aggregates in polymersomes for near-infrared photoacoustic imaging. ACS Appl Mater Interfaces.

[197] Kubelick KP, Emelianov SY (2020). Prussian blue nanocubes as a multimodal contrast agent for image-guided stem cell therapy of the spinal cord. Photoacoustics..

[198] Wang L, Xie S, Wang Z, Liu F, Yang Y, Tang C (2020). Functionalized helical fibre bundles of carbon nanotubes as electrochemical sensors for long-term in vivo monitoring of multiple disease biomarkers. Nat Biomed Eng.

[199] De La Zerda A, Zavaleta C, Keren S, Vaithilingam S, Bodapati S, Liu Z (2008). Carbon nanotubes as photoacoustic molecular imaging agents in living mice. Nat Nanotechnol.

[200] Cai K, Zhang W, Foda MF, Li X, Zhang J, Zhong Y (2020). Miniature hollow gold nanorods with enhanced effect for in vivo photoacoustic imaging in the NIR-II window. Small..

[201] Zhang H, Wang T, Qiu W, Han Y, Sun Q, Zeng J (2018). Monitoring the opening and recovery of the blood-brain barrier with noninvasive molecular imaging by biodegradable ultrasmall Cu(2- x)Se nanoparticles. Nano Lett.

[202] Wang Y, Hu X, Weng J, Li J, Fan Q, Zhang Y (2019). A photoacoustic probe for the imaging of tumor apoptosis by caspase-mediated macrocyclization and self-assembly. Angew Chem Int Ed Eng.

[203] Li Z, Fu Q, Ye J, Ge X, Wang J, Song J, et al. Ag(+) -coupled black phosphorus vesicles with emerging NIR-II photoacoustic imaging performance for cancer immune-dynamic therapy and fast wound healing. Angew Chem Int Ed Eng. 2020. 10.1002/anie.202009609. 10.1002/anie.202009609 32841465

[204] Jiang Y, Upputuri PK, Xie C, Zeng Z, Sharma A, Zhen X (2019). Metabolizable semiconducting polymer nanoparticles for second near-infrared photoacoustic imaging. Adv Mater.

[205] Lyu Y, Zeng J, Jiang Y, Zhen X, Wang T, Qiu S (2018). Enhancing both biodegradability and efficacy of semiconducting polymer nanoparticles for photoacoustic imaging and photothermal therapy. ACS Nano.

[206] Jiang Y, Upputuri PK, Xie C, Lyu Y, Zhang L, Xiong Q (2017). Broadband absorbing semiconducting polymer nanoparticles for photoacoustic imaging in second near-infrared window. Nano Lett.

[207] Taruttis A, Herzog E, Razansky D, Ntziachristos V (2010). Real-time imaging of cardiovascular dynamics and circulating gold nanorods with multispectral optoacoustic tomography. Opt Express.

